# Predicting Hemagglutinin MHC-II Ligand Analogues in Anti-TNFα Biologics: Implications for Immunogenicity of Pharmaceutical Proteins

**DOI:** 10.1371/journal.pone.0135451

**Published:** 2015-08-13

**Authors:** Benjamin J. Andrick, Alexandra I. Schwab, Brianna Cauley, Lauren A. O’Donnell, Wilson S. Meng

**Affiliations:** Division of Pharmaceutical Sciences, Duquesne University, Pittsburgh, PA, 15282, United States of America; National Institute of Infectious Diseases, JAPAN

## Abstract

The purpose of this study was to evaluate the extent of overlapping immunogenic peptides between three pharmaceutical biologics and influenza viruses. Clinical studies have shown that subsets of patients with rheumatoid arthritis (RA) develop anti-drug antibodies towards anti-TNFα biologics. We postulate that common infectious pathogens, including influenza viruses, may sensitize RA patients toward recombinant proteins. We hypothesize that embedded within infliximab (IFX), adalimumab (ADA), and etanercept (ETN) are ligands of class II major histocompatibility complex (MHC-II) that mimic T cell epitopes derived from influenza hemagglutinin (HA). The rationale is that repeated administration of the biologics would reactivate HA-primed CD4 T cells, stimulating B cells to produce cross-reactive antibodies. Custom scripts were constructed using MATLAB to compare MHC-II ligands of HA and the biologics; all ligands were predicted using tools in Immune Epitope Database and Resources (IEDB). We analyzed three HLA-DR1 alleles (0101, 0401 and 1001) that are prominent in RA patients, and two alleles (0103 and 1502) that are not associated with RA. The results indicate that 0401 would present more analogues of HA ligands in the three anti-TNFα biologics compared to the other alleles. The approach led to identification of potential ligands in IFX and ADA that shares sequence homology with a known HA-specific CD4 T cell epitope. We also discovered a peptide in the complementarity-determining region 3 (CDR-3) of ADA that encompasses both a potential CD4 T cell epitope and a known B cell epitope in HA. The results may help generate new hypotheses for interrogating patient variability of immunogenicity of the anti-TNFα drugs. The approach would aid development of new recombinant biologics by identifying analogues of CD4 T cell epitopes of common pathogens at the preclinical stage.

## Introduction

Tumor necrosis factor-alpha (TNFα) is a driving inflammatory mediator in rheumatoid arthritis (RA) [1]. RA patients benefit from anti-TNFα biologics through reduced disease activities and in some cases, remission [2]. Infliximab (IFX), adalimumab (ADA), both monoclonal IgG antibodies, and etanercept (ETN), a fusion protein, are the mainstay of the anti-TNFα biologics used in RA patients in the United States [3]. Despite the generally positive outlook in conferring long-term health benefits, approximately one-third of the patients receiving an anti-TNFα biologics do not respond to treatment [4]. Recent clinical studies have reported cases of persistent active diseases, despite continuing treatments at higher doses [5]. Such instances suggest potential drug neutralization by the immune system. A mechanistic understanding of the immunological basis underlying these phenomena will lead to improved treatment outcomes.

While multiple factors are implicated in driving therapeutic responses to anti-TNFα biologics in patients, a known cause of treatment failure is the development of anti-drug antibodies [5]. Such immunological reactions would accelerate drug clearance, resulting in sub-therapeutic plasma concentrations. IFX, ADA and ETN are recombinant proteins engineered to reduce intrinsic immunogenic potential. IFX is a chimeric IgG1-kappa monoclonal antibody with mouse variable regions grafted into human constant regions [6]. Bendtzen et al., however, reported that 44% of the 106 RA patients tested were found to have serum anti-IFX antibodies six months after initiation of treatment [7]. In some of these patients (13%), anti-IFX antibodies were detected as early as 1.5 months, or as few as after three infusions. Such antibodies are associated with low trough plasma drug concentrations, a metric predictive of poor efficacy. Among RA patients who tested positive for anti-IFX antibodies, Wolbink et al. reported fewer responders (36%) compared to patients without the antibodies (69%), [8]. The rapid development of antibodies in certain patients against IFX without inflammatory adjuvants suggests that prior environmental factors may raise the drug’s immunogenicity.

ADA is a “fully human” IgG1-kappa monoclonal antibody generated from in vitro screening of phage libraries displaying human variable regions [9]. Despite the lack of *bona fide* mouse sequences, anti-ADA antibodies have been detected in patients who have received the biologics. In a study that followed 272 RA patients for 156 weeks, Bartelds et al. reported that 28% of the patients tested positive for anti-ADA antibodies during the first 28 weeks of treatment [10]. The presence of such antibodies correlates with poor disease prognosis and secondary treatment failure. Importantly, assays used in these analyses were sufficiently specific to minimize interference by rheumatoid factors (RFs) [7]. Unlike IFX and ADA, ETN is a fusion protein consisting of the human tumor necrosis factor receptor-II (TNFRII) domain fused with human IgG1 constant Fc regions (CH2 and CH3). So far, studies have shown that prevalence of anti-ETN antibodies in patients is low [11–13]; Dore et al. have reported detecting non-neutralizing anti-ETN antibodies in 12 out of 214 RA patients [13].

Induction of anti-drug antibodies correlates with the presence of CD4 T cell epitopes presented by class II major histocomptability complex (MHC-II) alleles [14]. Antigen-presenting cells (APCs) would internalize and digest a biologic into small fragments. A subset of the trimmed peptidic fragments should bind to at least one MHC-II allele expressed in an individual. CD4 T cell clones that recognize the MHC/peptide complexes presented would in turn activate B cells to produce antibodies [15]. Thus, the repertoires of peptides selected by MHC alleles define the outer boundaries of anti-drug antibody responses. The variability of antibody response is in part a function of the diverse genetic make-up in humans; an individual’s CD4 T cell repertoire and HLA alleles shape the scope, magnitude, and kinetics of antibodies directed against a given biologic. The context in which the antigens are presented may be an overriding factor. Intense exposure to certain exogenous antigens may sensitize an individual towards a certain biologics. Cryptic epitopes in apparently non-immunogenic proteins may become stimulatory when the same, or similar sequences are presented in inflammatory milieus [16].

Viruses or other infectious pathogens may sensitize certain individuals to develop cross-reactive CD4 T cell responses in an MHC-II allele-dependent manner. The rationale is that embedded within immunogenic biologics are MHC-II ligands that share sequence homology with epitopes in the major antigens of the viruses. A systematic analysis of such ligand analogues could help gauge the risk for developing anti-drug antibodies in an allele-specific manner [17–19]. The presence of virus-primed helper T cells that recognize epitopes in anti-TNFα biologics would lower the threshold of drug-specific B cell activation. Consequently, anti-drug antibodies may arise sooner and to higher titers. This form of molecular mimicry may heighten the sensitivity to a given biologic in individuals exposed to certain viruses. It may contribute to the unexpectedly high number of RA patients who develop antibodies against anti-TNFα biologics after initiation of treatment, independent of intrinsic anti-IgG antibodies in circulation [20]. A recent meta-analysis by Lv et al. concluded that apparent association between RF and anti-TNFα therapeutic outcomes is not substantiated by published data [21], in congruent with that the incidence and magnitude of anti-drug antibodies vary greatly among individuals with RA [14].

In order to investigate potential cross-reactive viral epitopes, we focused on influenza type-A viruses because of widespread exposure in the population [22]. New influenza strains are introduced into the population due to seasonal variability. In the first global influenza pandemic in 40 years, the 2009 H1N1 pandemic strains largely displaced the viruses previously circulating in humans [23]. Since then the viruses have maintained a continual presence in North America through seasonal infections [24, 25], albeit with minor genetic changes in the major antigens such as hemagglutinin (HA). Cross-reactive memory CD4 T cells may be generated If HA antigens share similar MHC-II ligands in a given anti-TNFα biologics. These antigen-specific helper T cells would in turn activate B cells that produce antibodies against both the biologic and the viral HA.

**Fig 1:**
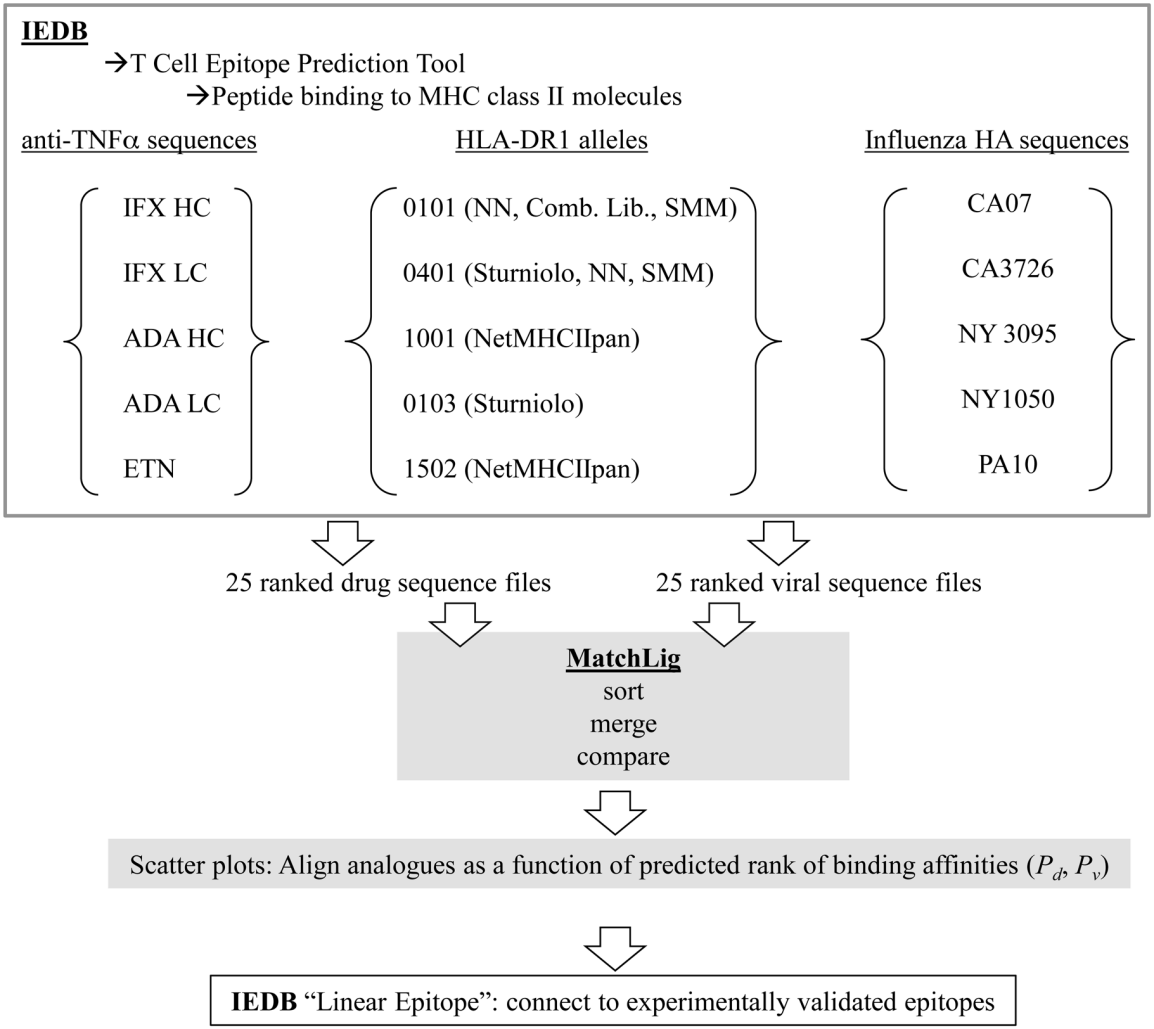
Schematic depiction of strategy used in identifying analogues of MHC class II ligands in HA sequences and anti-TNFα biologics. Analogues were identified from five anti-TNFα biologics polypeptides (heavy and light chains), five HLA-DR1 alleles, and five H1N1 influenza-HA sequences.

We describe herein a bioinformatics strategy to identify systematically potential cross-reactive T cell epitopes in HA and anti-TNFα biologics (Fig 1). We hypothesize that embedded within the primary sequences of IFX, ADA, and ETN are analogous sequences that resemble HA-derived MHC-II ligands. Such sequences in the biologics may stimulate naive or re-activate memory HA-specific T cells. Using IEDB and custom scripts generated using MATLAB, potential cross-reactive ligands, or analogues, were predicted for HLA-DR1*0101 (hereafter referred as 0101), HLA-DR1*0401 (0401), and HLA-DR1*1001 (1001), three MHC-II alleles that are implicated in RA [26]. Presumably, a higher proportion of the population who express one or more of these alleles would have been treated with at least one of the three anti-TNFα biologics. Also analyzed were HLA-DR1*0103 (0103) and HLA-DR1*1502 (1502) that are not associated with RA. Ligands of IFX, ADA, and ETN were matched against those ligands predicted from H1N1 influenza type A HA antigens. The results provide a potential molecular mechanism by which development of anti-drug antibodies may occur.

## Materials and Methods

We developed a strategy combining the predictive power of IEDB (http://www.iedb.org/) with custom scripts (S1 Fig) in searching for analogous sequences in a systematic manner (Fig 1). Potential MHC-II ligands in HA and anti-TNFα biologics were identified using the “Peptide Binding to MHC Class II Molecules” option under “T cell Epitope Prediction Tool” in IEDB. The function operates on validated public algorithms that integrate established structural criteria with experimental binding data. In all queries, we used the “IEDB recommended” option in which the “consensus approach” is typically selected; it combines results from at least three algorithms (NN-align [27], SMM-align [28], Sturniolo [29], NetMHCIIpan [27] and/or CombLib [30]). Peptides are scored based on the best median rank [30]. MHC molecules that lack at least three predictors are default to the NetMHCIIpan method [31]. Full descriptions of the scoring regime can be found on the IEDB web site (http://tools.immuneepitope.org/mhcii/help/#Method). Such percentile-based ranking has biophysical meanings insofar as relative strong binding affinities are correlated with low percentiles. For our analysis, the IEDB server selected the consensus method for 0101 and 0401, Sturniolo for 1502, and NetMHCIIpan for 1001 and 0103. Datasets of sequences generated from the IEDB predictions can be found in S5 Fig.

Custom scripts were created using MATLAB (released R2014b) to analyze the datasets generated from IEDB. The main script MatchLig.m (“MatchLig” hereafter) calls routines that compare (Compare.m), merge and sort sequences (S1 Fig). The scripts for merging and sorting were adopted from codes written by Van Loan and Fan [32]. Allele-specific ligands identified in HA were compared with ligands in each biologic predicted for the same MHC allele (Fig 1). Sequences of biologics and viral antigens used in the analysis were retrieved from NCBI and patent publications. Five influenza A viral HA sequences from North America were analyzed, with CA07 (accession# ACP41953.1) and NY3095 (accession# ACZ05293.1) isolated in 2009, CA3726 (accession# AIC73748.1) in 2014, PA10 (accession # ACA33735.1) in 2007 and NY1050 (accession # AHL89558.1) in 2006. For IFX [6] and ADA [33], the Fab regions containing the constant segments and variable segments in both heavy chain (HC) and light chain (LC) were queried. The constant segments in IFX and ADA share the same amino acid sequence (S2 Fig). For these two antibodies, only their Fab domains were analyzed because the junction between CH1 and CH2 in the heavy chains is common with human IgG1 antibodies. The entire sequence of ETN (accession# ABW59388.1 [34]), including fragments of TNFRII, and CH2 and CH3 domains of IgG1, was analyzed. Sequences with more than three overlapping amino acids to a given HA sequence were removed to reduce redundant sequences; overlapping sequences that mapped to different HA ligands were retained. The scripts were internally validated for consistency by confirming matching identities of sequences generated in the FASTA input and IEDB output (data not shown). Protein sequences were aligned using the Global Alignment (Needleman-Wunsch) function in the bioinformatics tool in MATLAB. Analyses were performed using Dell Optiplex or Macintosh Air computers.

In the context of the current study, “analogue” is defined as having at least 8 of the 15 amino acids being identical or similar (defined in Table 1) at each position. The threshold (53.3%) was chosen based on the assumption that side chains interacting MHC and TCR in a given bound peptide do not overlap. Four MHC-contacting side chains, or those pointing toward the floor of the binding groove, together define the binding motifs of the HLA alleles [35]. An additonal four or more upward orienting peptide side chains are seen in x-ray structures interacting with TCRs. Peptides with identical and similar amino acids at the same positions in the majority of the 15mer frame would likely to have similar bound conformations, thereby presenting conflating molecular surfaces to TCRs. The method does not discriminate amino acids in the central region or those near the termini. The threshold is justified further by examples of molecular mimicry of T cell epitopes in the literature in which similar degree of resemblance has been reported [36, 37]. To limit the scope of the analysis to the highly probably epitopes, we compared HA and biologics ligands up to the tenth percentile in binding affinities, the same affinity-related threhold used by Wang et al [30]. As such, for each HLA allele, 15-mers of IFX, ADA, and ETN were compared independently against 15-mers of each of the viral antigens.

**Table 1:** Definition of similar amino acids based on physiochemical properties^a^.

Acidic^b^	Basic^b^	Non-polar	Aromatic	Uncharged polar^c^
**D, E**	R,K	A, V, L, I, M	F, Y	S, T
				N, Q

^a^Acidic and basic amino acids have side chains that are ionized at pH 7.4. All natural amino acids are included except cysteine (C), proline (P), glycine (G), tryptophan (W)

^b^Ionized at neutral pH

^c^These amino acids contain hydrophilic side chains that are not ionized at neutral pH.

## Results

We used the predictive power of MHC-binding algorithms accessed through IEDB in identifying MHC-II ligands for three commonly used anti-TNFα biologics and influenza HA [38]. All MHC-II molecules share the same structural topology [39, 40]: a β-pleated sheet supporting two raised α-helices, which all together forming a peptide-binding groove. Select residues in the MHC binding groove constitute “pockets”, within which side chains of bound peptides are accommodated based on size and electrostatics. Unlike MHC class I molecules, the class II binding groove has an “open” configuration; theoretically there is no limit to the length of bound peptides, but typical ligands consist of 13–15 amino acids. Most of the MHC-peptide interactions take place within the binding groove, with MHC residues making contacts with typically a core of nine amino acids in the bound ligand [39]. The strategy in identifying MHC-II ligands is to “thread” the polypeptide (from N-terminal to C-terminal) through the experimentally validated motifs. The resultant peptides are ranked based on their predicted binding strengths relative to a pool of random sequences, expressed in percentile. We limited the analysis to peptides that ranked in IEDB within the 10^th^ percentile in relative binding strengths in order to eliminate sequences with less chance of specific binding.

The HA sequences CA07 and NY3095 of influenza A were selected because these strains were isolated during the 2009 H1N1 pandemic. They are now considered as part of the regular seasonal influenza infections because they are spread widely in the United States. Also included in the analysis were one strain isolated in 2014 (CA3726), one isolated in 2006 (NY1050) and one from 2007 (PA10). These sequences have varying degrees of differences in their amino acid sequences, with the two California strains more closely related than the others (S3 Fig). The HLA-DR1 alleles 0401, 1001, and 0101 have odds ratios for RA at 4.44, 4.22 and 2.17, respectively, which suggest correlations with the development of RA [26]. Also included were 1502 and 0103 that are associated with ulcerative colitis but not RA [26, 41]. Though a limited scope of sampling, these HA sequences and MHC alleles represent independent vectors that capture influenza strains across several years and genotypes in the population.

### Distribution of MHC-II ligands in anti-TNFα biologics

**Fig 2:**
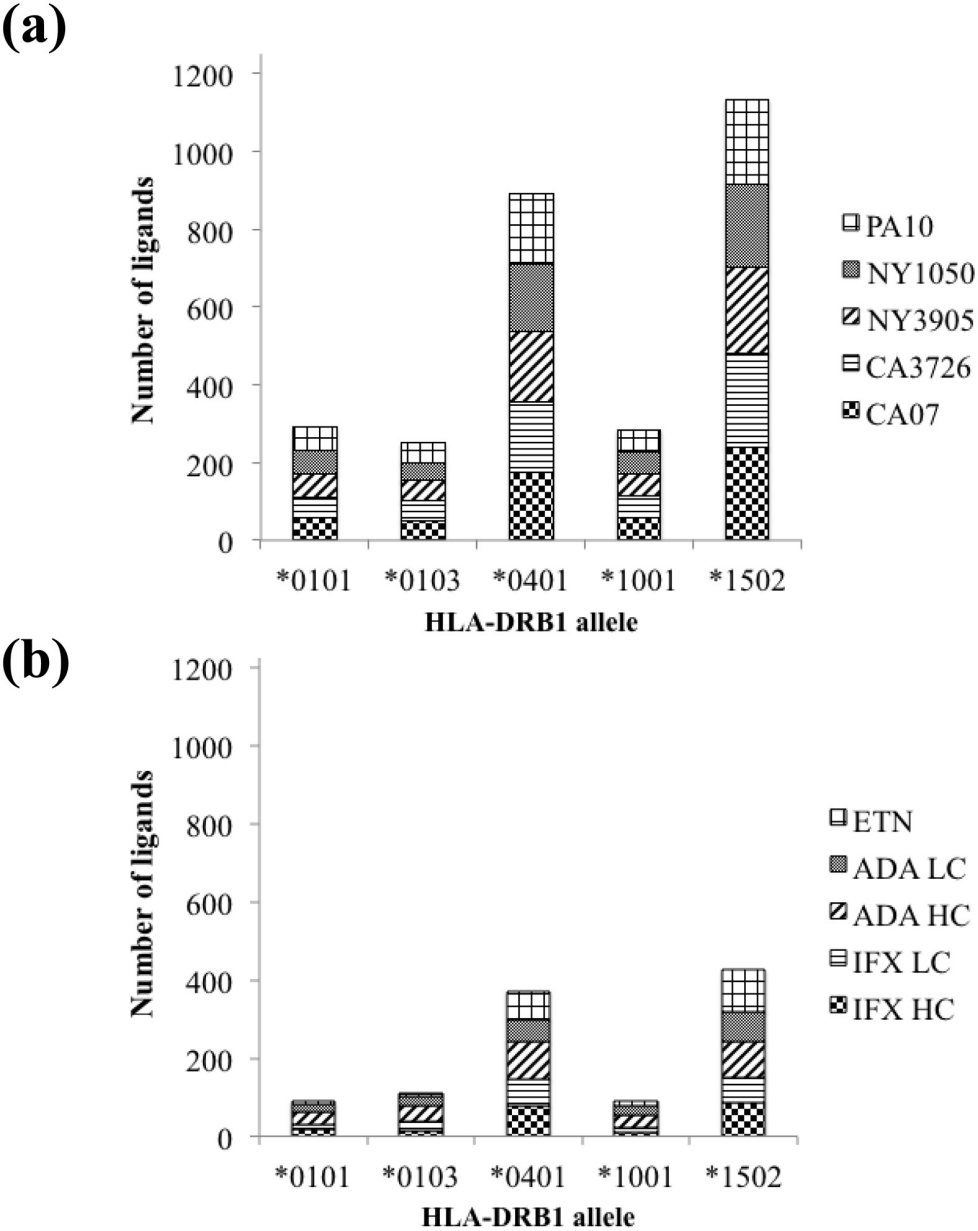
Distribution of IEDB-predicted ligands across five HLA-DR1 alleles in (a) selected HA sequences and (b) polypeptides of infliximab (IFX) and adalimumab (ADA) heavy and light chains, and etanercept (ETN). Only ligands ranked within the tenth percentile in binding strength are included. Locations of the ligands along the polypeptides are shown in S4a–S4c Fig.

The number of ligands generated in IEDB for the HA sequences and the anti-TNFα biologics are summarized in Fig 2. Only ligands that fell within the tenth percentile for a given allele are enumerated. For each allele, ligands are sectioned into HA (Fig 2a) or drugs (Fig 2b). The five HA sequences were analyzed for allele-specific ligands. Overall, more than three times as many ligands are predicted for 0401 and 1502 than in 0103, 0101 for each biologic (Fig 2b). These results suggest that 0401 and 1502 appear to accommodate more diverse amino acid side chains in the anchor positions compared to the other alleles. Because of their shared constant regions (S2 Fig), common ligands were found in IFX and ADA. An example is SSGLYSLSSVVTVPS (residues 179–193 in IFX and residues 180–194 in ADA) that is predicted to bind all three MHC alleles. Another common ligand is PAVLQSSGLYSLSSV, a peptide located in the constant region in IFX HC (residues 174–188) and ADA HC (residues 175–189). These sequences occupy a segment that has been shown experimentally as ligands of 0101 and 0401 [42, 43]. The ligand VSYLSTASSLDY, restricted by 0401 and 1001, is a sequence that spans the complementarity-determining region-3 (CDR3) of ADA HC. A six-amino acid thrombin-cleavable site separates the two domains of ETN. This junctional region, which spans from residues 201 to 251, is therefore unique and potentially immunogenic. However, no ligands in this region are ranked within the tenth percentile in MHC binding, although sequences are predicted to bind between 15–20 percentiles in 0101 and 1001 (data not shown).

### Scatter analysis of analogues

**Fig 3:**
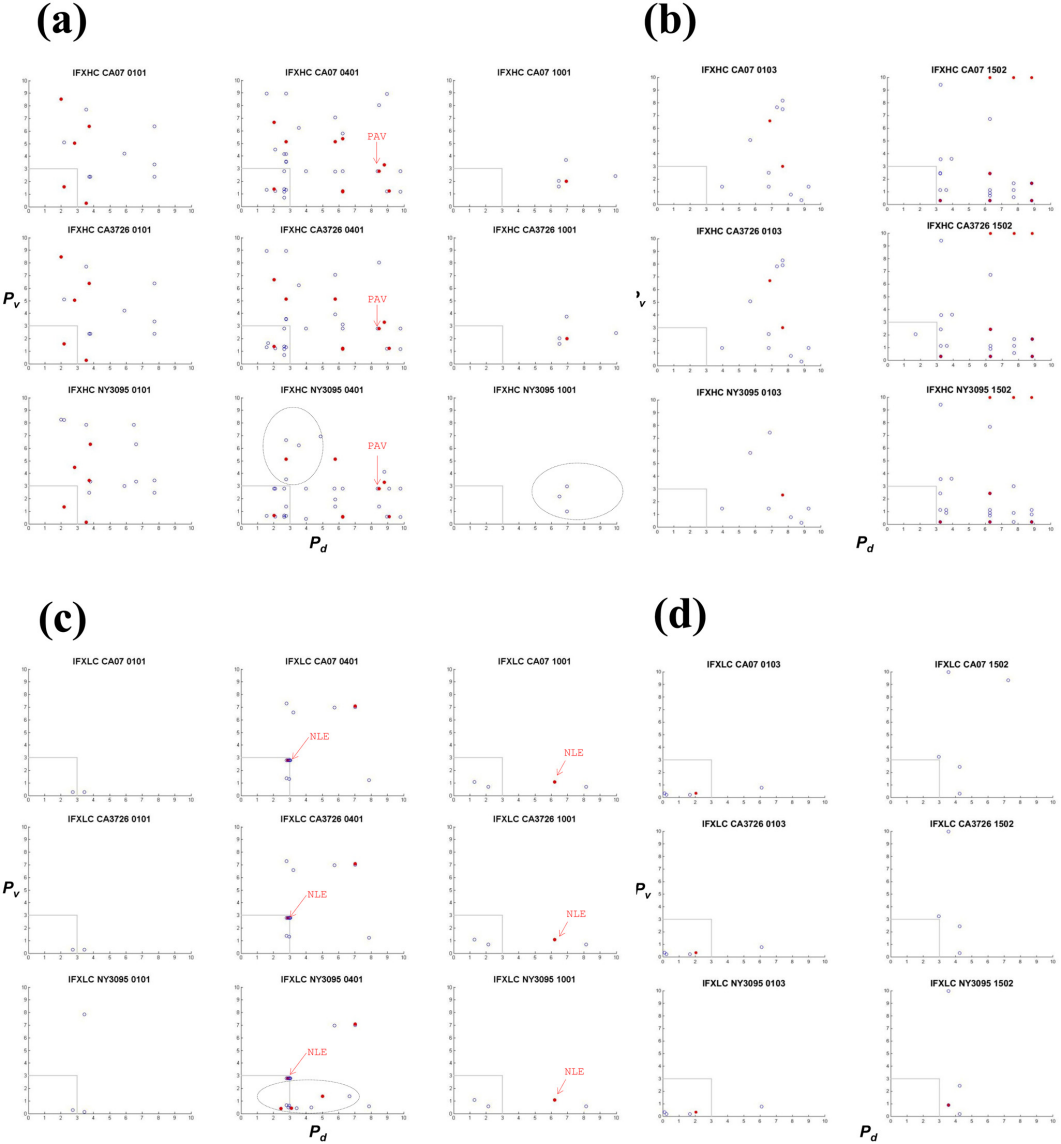
HA analogues in infliximab (IFX) heavy (HC) and light (LC) chains. The heavy chain was analyzed for (a) RA-associated HLA alleles (0101, 0401 and 1001) and (b) non-RA associated alleles (0103 and 1502). The light chain was also analyzed for (c) RA-associated HLA alleles (0101, 0401 and 1001) and (d) non-RA associated alleles (0103 and 1502). Matching biologic and viral ligand pairs are placed based on degrees of similarity and predicted relative binding strengths to the HLA allele. Each point represents a matching pair, identified based on their unique coordinates: the percentile ranking of a biologic sequence to the MHC allele (*P*_*d*_; x-axis), percentile ranking of the HA peptide homologous to the biologic sequence, to the same allele (*P*_*v*_; y-axis). Open blue circles represent biologic sequences that share 8 (out of 15) identical and similar amino acids (as defined in Table 1) with a HA ligand, whereas closed circles in red indicate pairs with at least 9 identical or similar amino acids. Arrows labeled “NLE” or “PAV” point to analogues in biologics that mimic the influenza CD4 T cell epitope HA_530–541_ (see also Table 3). Only the Fab regions were considered. Analyses for pre-2009 HA sequences (PA10 and NY1050) can be found in S4. Datasets containing the sequences can be found in S5 Fig

**Fig 4:**
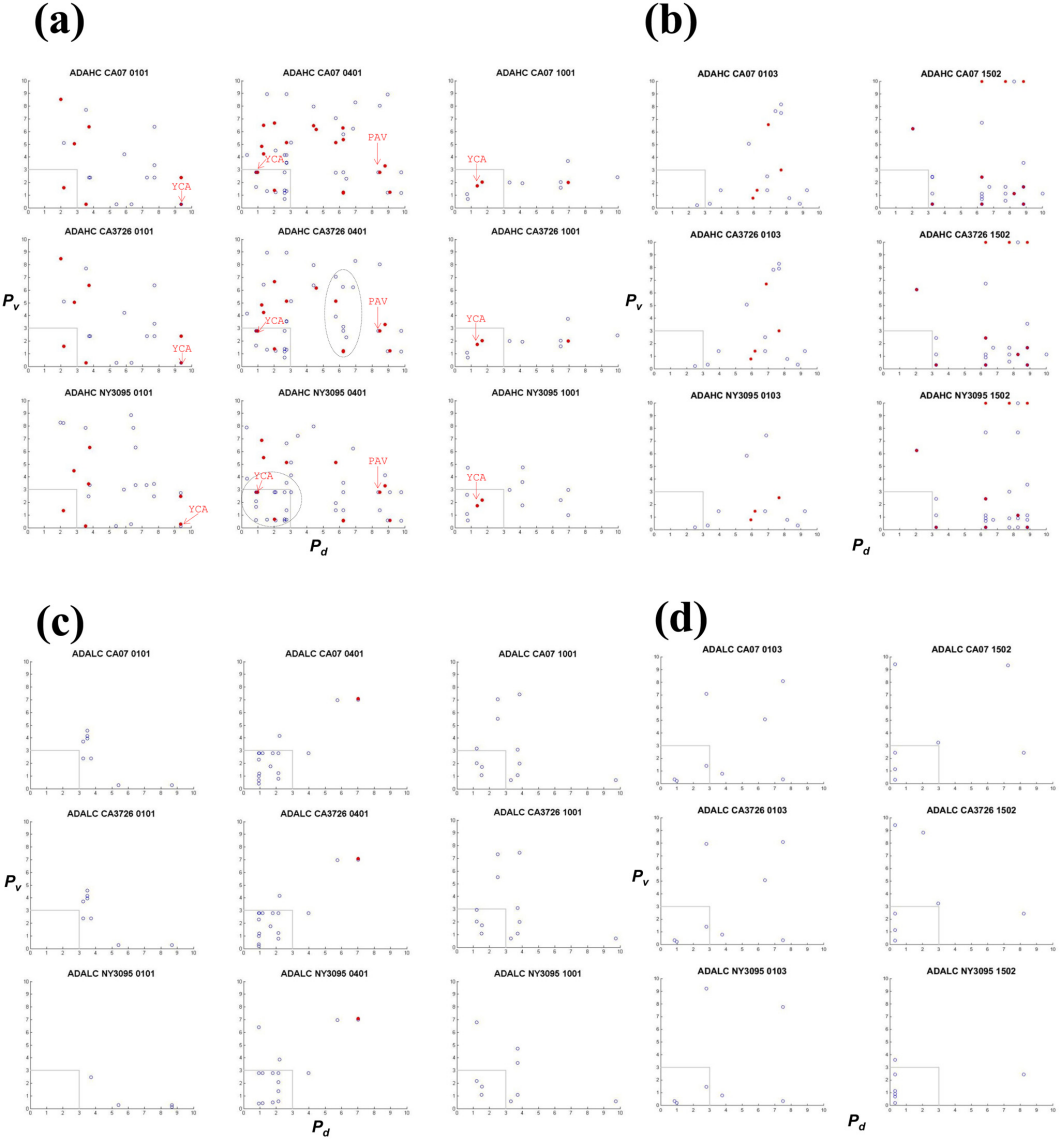
HA analogues in adalimumab (ADA) heavy (HC) and light (LC) chains. The heavy chain was analyzed for (a) RA-associated HLA alleles (0101, 0401 and 1001) and (b) non-RA associated alleles (0103 and 1502). The light chain was analyzed for (c) RA-associated HLA alleles (0101, 0401 and 1001) and (d) non-RA associated alleles (0103 and 1502). X axis and Y axis are defined in Fig 3 legend. Only the Fab regions were considered. Arrows labeled “YCA” or “PAV” point to analogues in biologics that mimic the influenza CD4 T cell epitope HA_530–541_ (see also Table 3). Analyses for pre-2009 HA sequences (PA10 and NY1050) can be found in S4 Fig Datasets containing the sequences can be found in S5 Fig.

**Fig 5:**
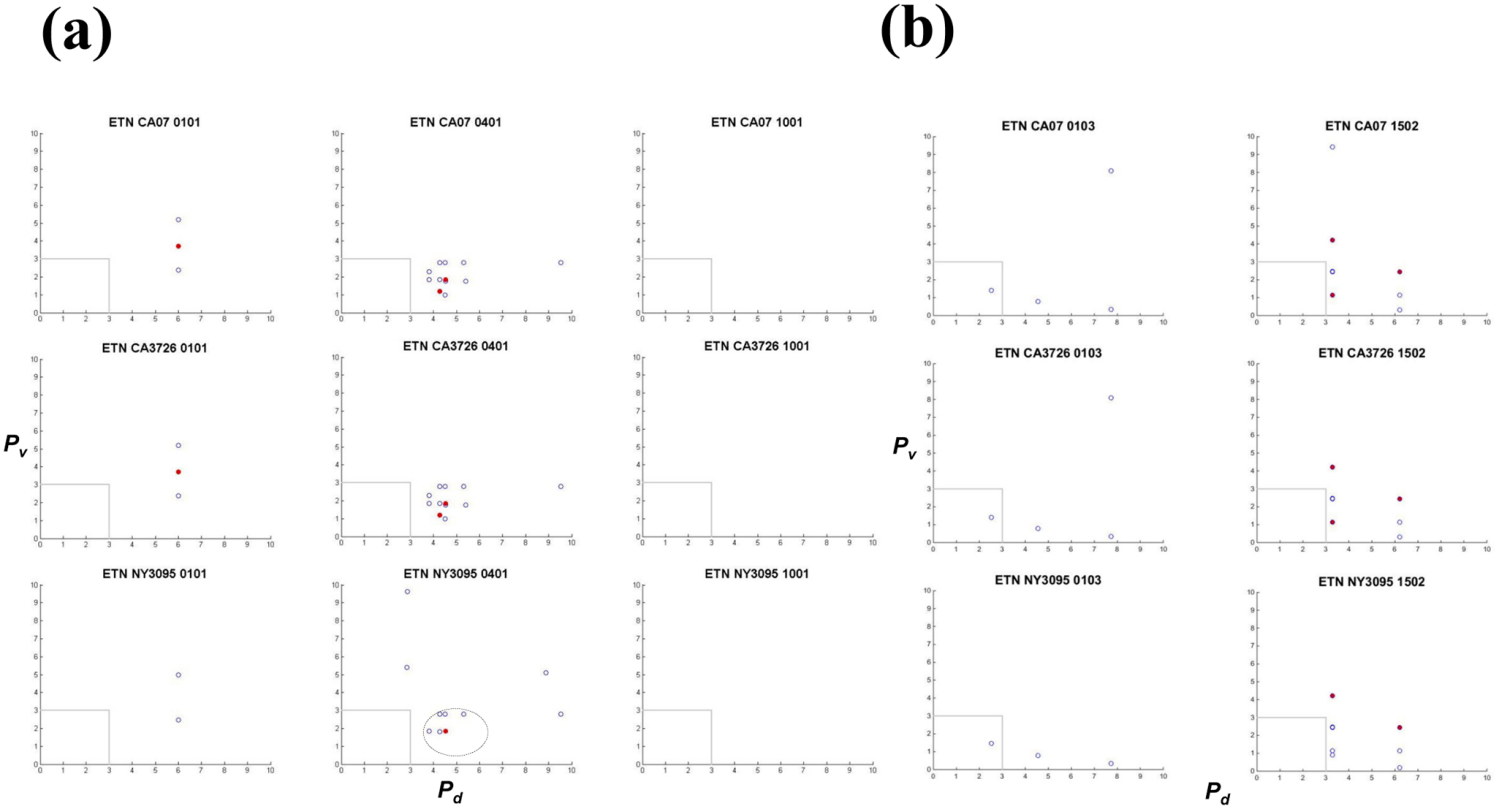
HA analogues in etanercept (ETN). The polypeptide was analyzed for (a) RA-associated HLA alleles (0101, 0401 and 1001) and (b) non-RA associated alleles (0103 and 1502). The entire polypeptide, inclusive of TNFR and IgG1 CH2 and CH3, were considered. X axis and Y axis are defined in Fig 3 legend. Analyses for pre-2009 HA sequences (PA10 and NY1050) can be found in S4 Fig Datasets containing the sequences can be found in S5 Fig

Analogues were extracted from ligands derived from the anti-TNFα biologics and HA sequences. An analogue in a given biologic is defined by three vectors: the percentile ranking of its own binding to the MHC allele (*P*_*d*_, x-axis), percentile ranking of its homologous HA peptide to the same allele (*P*_*v*_, y-axis), and the number of identical or similar amino acids at corresponding positions; open blue circles indicate analogues with 8 of the 15 residues matching, and red dots indicate those with at least 9 of the 15 matching). The results show that 0401 would present more analogues than the other alleles for all three biologics (Figs 3, 4 and 5 and Table 2). In ADA HC, 56 sequences bear homology with ligands in CA07 HA (Fig 4a). Of these biological ligands, 30 are ranked within the 3^rd^ percentile, suggesting exceptional high affinities for 0401. Of the same 56 sequences, 19 (red dots) contain more than nine (out of 15) identical or similar amino acids, including three with 10-amino acids homology (Table 2; S4 Fig). Four to five analogues were found with 1001 within the 3^rd^ percentile (Fig 4a), while only one in 0101 (Fig 4a) and 0103 (Fig 4b). None was found within the 3^rd^ percentile in 1502 (Fig 4b). The same pattern was found in pre-2009 sequences NY1050 and PA10 in that 0401 would present the most number of analogues (Table 2; S4 Fig). In ADA LC, 19 analogues are found restricted by 0401 (Fig 4c), with 15 ranked within the 3^rd^ percentile in binding, and one sharing nine identical or similar amino acids with an HA ligand. The allele 1001 would present the second most number of analogues in ADA LC among the alleles (Fig 4c), while 1502 would present the least (Fig 4d). While the number of potential ligands predicted for 0401 and 1502 are similar (Table 3 and Fig 2), only 0401 would present many more analogues within the 3^rd^ percentile (Figs 3, 4 and 5). Thus the analysis revealed an additional level of complexity.

**Table 2:** Comparison of HA ligand analogues in anti-TNFα biologics^a^.

Allele	IFX		ADA		ETN	HA sequence
	HC	LC	HC	LC		
0101	13^b^ (5^c^)	2(0)	19 (7)	9(0)	3(1)	CA07
	13 (5)	2(0)	19(7)	9(0)	3(1)	CA3726
	16 (5)	3(0)	24 (7)	4(0)	2(0)	NY3095
	17 (6)	3(0)	26 (8)	4(0)	2(0)	NY1050
	16 (5)	3(0)	25 (7)	4(0)	2(0)	PA10
0401	39 (11)	14 (2)	56 (19)	19 (1)	12 (2)	CA07
	35 (10)	14 (2)	51 (15)	19 (1)	12 (2)	CA3726
	36 (9)	19 (5)	54 (13)	18 (1)	10 (1)	NY3095
	38 (10)	19 (5)	57 (14)	17 (1)	7 (1)	NY1050
	36 (9)	19 (5)	55 (13)	18 (1)	10 (1)	PA10
1001	5(1)	4(1)	11 (3)	12 (0)	0(0)	CA07
	5(1)	4(1)	11 (3)	12 (0)	0(0)	CA3726
	3(0)	4(1)	13 (2)	9(0)	0(0)	NY3095
	5(1)	4(1)	15 (3)	8(0)	0(0)	NY1050
	3(0)	4(1)	13 (2)	9(0)	0(0)	PA10
0103	12 (2)	5(1)	16 (4)	8(0)	4(0)	CA07
	12 (2)	5(1)	16 (4)	8(0)	4(0)	CA3726
	8 (1)	5(1)	12 (3)	7(0)	3(0)	NY3095
	9 (2)	5(1)	14 (4)	6(0)	3(0)	NY1050
	8 (1)	5(1)	12 (3)	7(0)	3(0)	PA10
1502	42 (10)	7(0)	47 (13)	15 (0)	21 (4)	CA07
	44 (10)	6(0)	47 (13)	15 (0)	20 (4)	CA3726
	44 (9)	11 (3)	52 (12)	13 (0)	15 (3)	NY3095
	48 (10)	11 (3)	56 (13)	11 (0)	15 (3)	NY1050
	44 (9)	11 (3)	52 (12)	13 (0)	15 (3)	PA10

^a^Corresponds in part to datasets S4 and S5 Figs.

^b^Total number of analogues mapped (8 of 15 identical or similar amino acids) to HA ligands; biologic and viral ligands are ranked within the 10^th^ percentile were tallied

^c^Number of sequences containing at least 9 (out of 15) identical or similar amino acids mapped to a HA ligand.

**Table 3:** Highly similar sequences in biologics mapped to the known CD4 T-cell epitope HA_530–541_ ILAIYSTVASSL^a^.

Corresponding anti-TNFα biologic	Biologic (top) and viral (bottom) sequences and their alignment (shown in middle)^b^	Percentile rank within allele					Corresponding HA strain
		0101	0401	1001	0103	1502	
IFX LC_103–117_ ^c^	NLEVKRTVAAPSVFI		2.86	6.21	2.05		
	Qi*a**s*vA*SI*I	consensus					
	QILAIYSTVASSLVL		2.79	1.09	0.33		CA07_529–543_ CA3726 _529–543_ NY3095_528–542_ NY1050_528–542_ PA10_528–542_
ADA HC_95–109_ ^c^	YCAKVSYLSTASSLD	9.36	0.89	1.37	5.39	8.27^d^	
	Y * i * a * Y * t * ASSL *	consensus					
	YQILAIYSTVASSLV	0.28	2.79	1.72	0.79	1.13	CA07_528–542_ CA3726_528–542_ NY3095_527–541_ NY1050_527–541_ PA10_527–541_
IFX HC_174–188_/ ADA HC_175–189_	PAVLQSSGLYSLSSV		8.46			6.27	
	* lai * St * a * SL**V	consensus					
	ILAIYSTVASSLVLV		2.79			2.45	CA07_530–544_ CA3726_530–544_ NY3095_529–542_ ^e^ NY1050_529–542_ ^e^ PA10_529–542_ ^e^

^a^Reported by Schanen et al. in *Vaccine* 29: 3299–3309 [44].

^b^Only analogues with at least 9 of the 15 amino acids being identical or similar are shown. Biologic sequences were mapped to the viral epitope using the IEDB “Linear Epitope” search tool set at “70% blast”. Identical and similar amino acids are indicated in the consensus sequence, with upper case indicates identical, lower case indicates similar, “*” indicates neither. These sequences are indicated with arrows in Figs 3 and 4 and can be found in dataset S5 Fig ETN analogues met the criteria were not found.

^c^Both peptides are unique to the biologics (S2 Fig).

The “NLE” peptide in IFX LC spans across the junction between the variable and constant region, while the “YCA” peptide in ADA HC is located in the CDR-2.

^d^In ADA HC, a slightly shifted sequence, YYCAKVSYLSTASSL (residues 94–108), is mapped to the viral epitope

^e^The last amino acid is a leucine in NY3095, NY1050, and PA10.

Likewise, in IFX and ETN, 0401 would present more analogues than the other alleles (Figs 3 and 5). In IFX HC, 39 sequences bear homology to HA-derived 0401 ligands (Fig 3a and Table 2). Of the 39, 11 share more than nine identical or similar amino acids with a CA07 ligand, and 19 that are ranked within the 3^rd^ percentile in binding to 0401. IFX HC (Fig 3a and 3b) and ADA HC (Fig 4a and 4b) contain more analogues compared to their respective light chains (Figs 3c, 3d, 4c and 4d). Overall, fewer analogues are predicted in ETN (Fig 5): 12 with 0401, three with 0101, and none with 1001. But six to seven were found with 1502 in both post- and pre-2009 HA sequences (Fig 5b and S5 Fig). Collectively, the scatter analysis provides qualitative means for comparing cross-reactivity of the biologics across the five MHC alleles.

Several observations were made with regard to the five influenza HA sequences. Overall, substituting CA07 with NY3095 does not alter the general scattering pattern (Table 2). On the other hand, NY3095 (2009) renders unique analogues that are absent in CA07 (2009) and CA3726 (2014). For example, more analogues are located below the 3^rd^ percentile region (dotted area) in NY3095 and IFX LC for 0401 compared to the same regions in CA07 and CA3726 (Fig 3c). Other NY3095 unique analogues are also detected in the IFX HC (0401 and 1001), ADA HC (0101, 0401, and 1001), ADA LC (0101, 0401, and 1001) and ETN (0401). NY3095, NY1050, and PA10 shares 80% identity with CA07, whereas CA07 and CA3726 share 98% identities (S3 Fig). Despite the relatively high degrees of identifies, dissimilarities between the California strains and the New York (and Pennsylvania) strains are manifested in the extent to which their ligands overlap (S5 Fig: m, n and o). The top three panels show scatter plots of matching CA07 against CA07 for the same MHC allele (S5 Fig: m). Symmetry is indicated by the red dots along the centerline between x and y axis, because the same sets of ligands are matched. Ligands of CA07 and CA3278 closely overlap. A high degree of symmetry exists between NY3095 and CA07 for 0401, but no symmetry can be seen in 0101 and 1001 between the two HA sequences. In comparing pre-2009 to post-2009 HA analogues, only 0401 shows some degrees of symmetry, for example between CA07 and NY1050, and CA07 and NY1050 (S5 Fig: n and o). These analyses served as internal check of the scripts and provide a sensitive way to compare closely related sequences with respect to antigenicity.

### Analogues of a dominant HLA-DR1-restricted HA epitope

We extended the scatter analysis by identifying analogues that are more likely to cross-react with HA epitopes. This was carried out by searching in IEDB (using “Linear Epitope” search tool at “70% blast”) for known HA-derived CD4 T cell epitopes that share homology with the analogues. The analysis identified sequences in IFX and ADA that encapsulate the HA peptide ILAIYSTVASSL (residues 530–541) conserved across all five HA sequences analyzed. This MHC-II restricted epitope was discovered in humans exposed to 2009 strains of swine-origin influenza (H1N1) [44] and induces strong CD4 T cell responses [44]. It is identified as viral ligands of 0401 (*P*_*v*_ = 2.79) and 1001 (*P*_*v*_ = 1.09) in IEDB. Table 3 highlights sequences that have at least nine of the 15 amino acids that are identical or similar to HA_530–451_. Analogues are found in the antigen-binding regions in heavy and light chains of IFX and ADA but not in ETN (Table 3).

IFX LC contains the analogue NLEVKRTVAAPSVFI (residues 103–117) that spans the junction (at residues 109 and 110) between the variable (V1) and constant (CH1) domains (Table 3). This sequence (Fig 3c, labeled “NLE”), unique to IFX, is predicted as high affinity ligands of 0401 and 1001. Two ADA LC sequences in the same region, VEIKRTVAAPSVFIF (residues 104–118) and KVEIKRTVAAPSVFI (residues 103–117), are mapped to the HA peptide ILAIYSTVASSLVLV, with 8 identical/similar amino acids out of the 15, it is not listed in Table 3. The ADA LC peptides are both ranked at 1.79 percentile in 0401. The ADA HC peptide YCAKVSYLSTASSLD (residues 95–109) is also an analogue of HA_530–451_ and is predicted as ligands of 0101, 0401, and 1001. This peptide, labeled in Fig 4c as “YCA”, spans the complementarity-determining region 2 (CDR-2) region (residues 99–110) in ADA HC [33], thus unique to the biologic. Using IEDB, YAC is partially mapped to ASQKRPSQRHGSKYLATAST, an HLA-DR-restricted MBP epitope implicated in multiple sclerosis in humans [45, 46]. The clinical implications of this match, however, remain to be investigated. Lastly, the sequence PAVLQSSGYSLLV in IFX HC (residues 174–188) and ADA HC (residues 175–189), known ligands of several HLA-DR1 alleles [42, 43], is identified as an HA_530–451_ analogue presented by 0401 (Table 3). Located in the constant CH1 region, this common analogue is indicated in Fig 3a and Fig 4a as “PAV”. The analysis illustrates an approach in which candidate ligands can be triaged using IEDB based on homology to known epitopes.

### A potential HA-cross reactive B cell epitope in adalimumab

The positional relationship of helper T- and B-cell epitopes may influence immunogenicity [47, 48]. Peptides in which a T cell epitope overlaps with a B cell epitope tend to generate antibodies of higher affinities than co-immunization of positional-separated epitopes. Thus we searched using IEDB to determine if analogues in the anti-TNFα biologics overlap with B cell epitopes. We mapped an ADA analogue to a known HA-derived B cell epitope. The ADA peptide, AKVSYLSTASSLDYW, located in the CDR-3 region (residues 97–111), shares homology to the B cell epitope core LSTASSWSY (HA_86–92_) discovered in humans exposed to the influenza viruses [49]. The core is located in CA07 and CA3726 but not in the other three sequences analyzed; NY3095, PA10 and NY1050 all have the “STAS” tract replaced by “ISKE” (S3 Fig), rendering different electrostatic properties.

**Fig 6:**
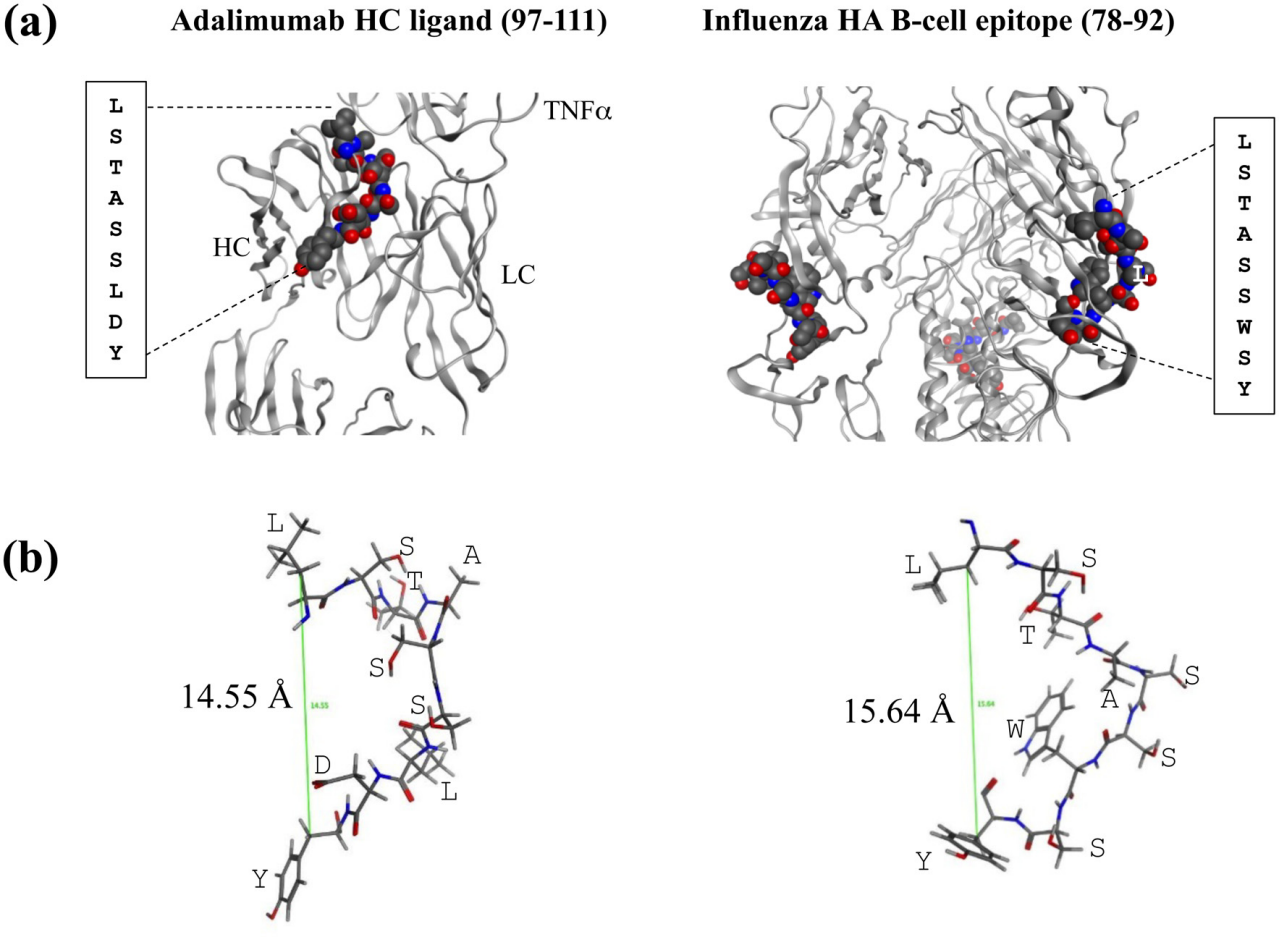
(a) Molecular representation of an ADA analogue that resembles a known B cell epitope in HA Zhao et al. (2011) [49]. The ADA CDR3 peptide was mapped to the B cell epitope by searching for known epitopes in IEDB. Images were generated using Molecular Operating Environment (MOE). Crystal structures of HA (PDB entry: 4M4Y, residues 174–182) and ADA (PDB entry: 3WD5, heavy chain residues 102–111) were used in the modeling. (b) The distances between the Cβ of the first and last amino acids are 14.55 and 15.64 Angstroms in ADA HC and HA, respectively. Locations of the side chains were labeled with single amino acid letter codes.

Patients exposed to H1N1 influenza viruses during and since the 2009 pandemic would likely have circulating anti-HA_78–92_ antibodies [49]. While it remains to be determined if such antibodies can cross-recognize LSTASSLDY on ADA, it should be pointed out that crystal structures show the two fragments adapt similar conformations (Fig 6). Both sequences adopt outward bend conformations with distances between the Cβ atoms of the first and last residues being 14.55 Å and 15.64 Å, respectively (Fig 6b). Thus generation of ADA-reactive antibodies may be more efficient than expected, as repeated intradermal administrations of ADA would be akin to booster vaccinations with AKVSYLSTASSLDYW in which helper T cell and B cells are reactivated concomitantly. The ADA peptide is predicted as high affinity ligands of 0401 (*P*_*d*_ = 1.37) and 1001 (*P*_*d*_ = 0.89), and as having a good chance of being presented. Memory HA_78–92_-specific B cell clones could internalize ADA via LSTASSLDY and could present AKVSYLSTASSLDYW to naïve CD4 T cells restricted by 0401 or 1001. HA-primed CD4+ T cells could also recognize AKVSYLSTASSLDYW; it shares 8 of 15 amino acids with in HA residues 87–101 (CA07) and binds 0401 at 11.54 percentile (thus not included in our scatter analysis). With 1001, the segment 83–97 that encompasses LSTASSWSY is predicted to bind at 30.09 percentile, a relatively weak binding peptide. These scenarios illustrate a strategy in which potential 0401 ligands in biologics can be selected for experimental testing.

## Discussion

Plasma drug concentration predicts the success of anti-TNFα biologics in RA patients [12, 50]. The advent of specific assays that exclude interferences by anti-IgG antibodies helped establish that clearance of IFX, ADA, and ETN is a function of anti-drug antibody response [4]. Given that all recombinant proteins are potentially immunogenic, we investigated a scenario in which elimination of the biologics is accelerated through exposure to influenza viruses. Patients exposed to influenza viruses could harbor memory lymphocytes that produce cross-reactive antibodies upon repeated exposure to biologics. A key variable is the vast number of HA sequences in the population; influenza viruses undergo sequence variations over time, leading to emergence of strains that are temporally unique [23]. Generation of anti-drug antibodies may be enhanced by the presence of helper T cells that recognize HA epitopes homologous with drug ligands restricted by HLA-DRB1 alleles.

We used a stepwise triaging approach in identifying sequences in biologics by simultaneously querying multiple HA sequences. IEDB generated datasets were systematically analyzed based on degrees of homology and relative binding strengths using custom Matlab scripts. We report analogues of HA-derived MHC-II ligands may embed within IFX, ADA, and ETN. Analogues include several peptides spanning regions unique to the biologic: variable-constant junctions and CDRs (Table 3 and Fig 6). The number and diversity of analogues varied by HLA haplotype, with no correlation between the strength of the odds ratio to develop an autoimmune disease and the number of analogues found. The data suggest 0401 would present more HA-mimicking analogues compared to the other four alleles analyzed (Fig 4). The number of biological ligands predicted in IEDB for 0401 and 1502 (Fig 2b) are similar, and more than each of the other three alleles. This comparison might suggest the two alleles carry more or less similar immunogenic risk for the anti-TNFα biologics. Our cross-reactivity analysis, however, reveals that only 0401, but not 1502, would present high affinity biologic ligands that mapped to high affinity viral ligands. For 0401, the 3^rd^ percentile regions across all HA sequences in both IFX and ADA (including HC and LC) are well populated (Figs 3 and 4). Conversely, for 1502, ADA LC contains three or four analogues in the 3^rd^ percentile region, with one high affinity analogue found in IFX HC mapped to a peptide in CA3726. These results highlight 0401 as a potentially unique HLA allele that warrants further analyses in relating IFX and ADA treatment outcomes with history of influenza infections.

The analysis focused on an underexplored relationship between biologics and viral immunity. But cross-reactivity of viral immunity has been well documented in several diseases. Promiscuous CD4 T cells may contribute to H1N1 immunity to greater extent than previously thought [44]. Treatment outcomes in RA patients being managed with Disease-Modifying Anti-rheumatic Drugs (DMARDs) correlate with previously acquired immunity to cytomegalovirus (CMV) [51]. The severity of joint destruction in RA correlates with the degree of preexisting immunity to CMV and EBV [52, 53]. Cross-recognition of self-antigens in autoimmune diseases can be precipitated by specific viral infections [54]. The Coxsackie B virus is implicated in eliciting autoimmune myocarditis and Type I diabetes [55, 56]. Systemic lupus erythematosus is associated with high titers of anti-Epstein-Barr virus (EBV) antibodies in affected joints and skin [57]. Multiple sclerosis may originate in some cases by cross-recognition of EBV-specific T cells with an epitope in myelin basic protein (MBP) restricted by HLA-DRB1*1501 [58]. The MBP epitope, ENPVVHFFKNIVTPR (residues 85–99), shares sequence homology with the immunodominant EBV peptide, TGGVYHFVKKHVHES [59]; seven of the 15 amino acids are identical or similar with respect to their physiochemical properties. The extent of homology, seven of fifteen identical or similar amino acids, between the viral and MPB epitopes in this documented case support the criterion that only sequences that share at least eight of the fifteen amino acids are considered as analogues.

Generation of anti-drug antibodies can be influenced by a multitude of drug, patient, and environmental factors. With respect to drug product quality, differences in glycosylations may lead to recogition of a biologic by antibodies. Impurities associated with manufacturing processes (e.g. adventitious viruses) can act as adjuvant, thereby boosting an apparent non-immunogenic protein to become immunogenic by inducing local inflammation[14]. Altered protein structures and conformations resulting from physical (e.g. denaturation, aggregration) and chemical (e.g. deamination, oxidation) damages are associated with increased immunogenicity. Duration, frequency, and route of adminstration are other factors implicated. Though it is not yet feasible to unify these interactive factors for a priori determination [14, 60, 61], a common criterion is that the drug polypeptide should contain epitopes of T and B cells.

The analysis has several limitations. We focused on matching primary sequences of MHC ligands in the biologics and HA from the influenza virus, recognizing that mere presentation by MHC molecules is not sufficient for predicting a T cell response. Similarities between primary sequences do not necessarily predict cross-reactivity; a bound ligand should mimic the conformation and chemical surface of a known epitope for T cell mimicry to occur. Non-conforming amino acids can override the conformational contributions of the identical or similar amino acids [62]. With this in mind, we mapped analogues to a known epitope in HA and identified several cross-reactive candidates in IFX and ADA. These analogues appear to mimic HA_530–541_, a promiscuous binder to HLA-DRB1 alleles [44]. The analysis also led to discovery of a core sequence in the B cell epitope HA_78–92_ embedded in the CDR3 region of ADA. The 15-mer ADA-derived peptide AKVSYLSTASSLDYW, containing both T and B cell epitopes, may be useful as a tool for screening patients for anti-ADA antibodies. But only CA07 and CA3726 contain the core LSTASSWSY; the other three HA sequences have the “STAS” tract broken by “ISKE”, rendering cross-reactivity of these strains with ADA-specific B and T cells unlikely. Thus it may be important to specifically identify the strain of HA in analyzing cross-reactivity between influenza and ADA.

The tenth percentile cutoff may be a conservative limit; ligands ranked above the threshold may render cross-reactivity. The datasets likely have omitted sub-dominant epitopes, given the degeneracy of T cell recognition [63]. The magnitude of the response would depend on the quality and frequency of memory T and B cells. Responses toward ligands with intermediate binding affinities may be amplified from repeated injections. The abundance of the ligands are not considered in our analysis, but anti-TNFα biologics administered through subcutaneous and intramuscular routes are akin to vaccinations without inflammation-inducing adjuvant. Another consideratin is that some MHC-II ligands, even high affinity ones, may be tolerogenic to T cells rather than stimulatory [42, 43]. Denatured biologics tend be more immunogenic [64], partly because protein aggregates are taken up by APCs more efficiently than native, soluble proteins. And chemical degradation can modify the antigenicity of innocuous proteins, resulting in altered binding affinity to MHC and recognition by TCRs.

Despite these caveats, the analysis generated sequences in anti-TNFα biologics that warrant further analyses. The strategy can be used in optimizing clinical studies aimed at understanding the variability of anti-drug antibodies in RA patients; MHC alleles and exposure to viral may be included as weighed variables. Select analogues may serve as molecular tools for predicting non-responders, and may be eliminated in engineering new biologics [65, 66]. The custom script MatchLig can be modified for analysis of proteins of other pathogens that may predispose anti-drug antibodies toward biologics. The principle lies in authenticating the relevancy of analogues by mapping to known T cell epitopes using IEDB, a dynamically updated public resource.

As of early 2015, IEDB archives more than 130,000 peptidic epitopes, including more than 30,000 T cell epitopes validated experimentally. Using this strategy, several biologic-derived analogues were mapped to HA_530–541_, an immunodominant epitope validated in human PBMC. In addition, an analogue we identified in the CDR3 region of ADA is mapped to a B cell epitope in HA (residues 86–92).

## Conclusion

Immunogenicity is a critical quality attribute of pharmaceutical biologics. The present paper demonstrates an approach for analyzing T cell cross-reactivity of biologics and HA, a major antigen of a common virus. The methods enhance the analysis of data retrieved from IEDB by systematic treatments of multiple viral strains, MHC alleles, and biologics. The analogues generated represent candidate cross-reactive epitopes prioritized for testing in T cell response assays. The datasets can be triaged further by mapping analogues to known T and B cell epitopes, as shown in Table 3 and Fig 6. These original findings thus illustrate the overarching essence of our approach: dozens (or more) MHC ligands predicted for a given pharmaceutical biologic can be narrowed to a few candidates interlinked with experimental data.

## Supporting Information

S1 Fig Matlab scripts: MatchLig.m and Compare.m. (DOCX)

S2 Fig Global alignment of infliximab and adalimumab using the Needleman-Wunsch algorithm via the function ‘nwalign’ in MATLAB Bioinformatics tools. “*” indicates junctions between variable and constant regions in the polypeptide. Boxed regions indicate HA ligands that are mapped to biologic ligands (Table 3).
(PDF)

S3 Fig Global alignment of HA strains using the Needleman-Wunsch algorithm via the function ‘nwalign’ in MATLAB Bioinformatics Tools, with CA07 as reference for comparison. The purple box in CA07-CA3726 enclosed the core sequence of B and T epitopes mapped to ADA HC CDR-3 (Fig 6). The core LSTASSWSY is broken in NY3095, NY1050, and PA10. Boxed regions in blue indicate common analogues, biologic sequences mapped to viral ligands (Table 3). Despite the >80% identities, cross-matching CA07 with ligands of the four strains (using MatchLig), S4 Fig (m-o) shows different patterns of ligands for all alleles except 0401 (with respect to their percentile rank in binding).
(PDF)

S4 Fig Predicted ligands placed along the polypeptides of (a) IFX, (b) ADA, and (c) ETN. Analogues in pre-2009 H1N1 influenza HA for five HLA-DR1 alleles: (d) IFX heavy chain presented by alleles associated with RA, (e) and not associated with RA; (f) IFX light chain presented by alleles associated with RA, (g) and not associated with RA; (h) ADA heavy chain presented by alleles associated with RA, (i) and not associated with RA; (j) ADA light chain presented by alleles associated with RA, (k) and not associated with RA; (l) ETN presented by alleles associated with RA, (m) and not associated with RA (j). (n), (o), and (p) show cross-matching of ligands between four HA sequences against CA07. *P*_*d*_ denotes percentile ranking of a given ligand in a biologic, and *P*_*v*_ denotes percentile ranking of a given ligand in a viral HA sequence.
(PPTX)

S5 Fig Analogue sequence datasets generated using MatchLig in Excel spreadsheets. (ZIP)
