# Supplementary material for: Predicting Hemagglutinin MHC-II Ligand Analogues in Anti-TNFα Biologics: Implications for Immunogenicity of Pharmaceutical Proteins
Source: PLoS One. 2015 Aug 13;10(8):e0135451. doi: 10.1371/journal.pone.0135451 (PMC4536234; doi:10.1371/journal.pone.0135451)
Supplement: S1 Fig — (DOCX) [file pone.0135451.s001.docx]

**MatchLig.m**

dname = input('File name of drug: ','s');

vname = input('File name of flu virus: ','s');

title1 = input('Title of graph and excel output: ','s');

AA = input('Which AA to eliminate (1 letter)? Enter ''X'' to keep all results: ','s');

AA = upper(AA);

[vPer,~] = xlsread(vname,'G:G');

[vIdxA,~] = xlsread(vname,'C:C');

[vIdxB,~] = xlsread(vname,'D:D');

vlen = length(vPer);

vRange = ['E2:E' num2str(2+vlen-1)];

[~,vPep] = xlsread(vname,vRange);

V=struct;

i=1;

while (i <= vlen) && (vPer(i) <= 10)

V(i).idxA = vIdxA(i);

V(i).idxB = vIdxB(i);

V(i).peptide = vPep(i);

V(i).percentile = vPer(i);

i = i+1;

end

[dPer,~] = xlsread(dname,'G:G');

[dIdxA,~] = xlsread(dname,'C:C');

[dIdxB,~] = xlsread(dname,'D:D');

dlen = length(dPer);

dRange = ['E2:E' num2str(2+dlen-1)];

[~,dPep] = xlsread(dname,dRange);

D=struct;

i=1;

while (i <= dlen) && (dPer(i) <= 10)

D(i).idxA = dIdxA(i);

D(i).idxB = dIdxB(i);

D(i).peptide = dPep(i);

D(i).percentile = dPer(i);

i = i+1;

end

figure

hold on

pt1 = [0,3]; pt2 = [3,3];

col = [0.8 0.8 0.8];

plot(pt1,pt2,'color',col,'LineWidth',1.5)

plot(pt2,pt1,'color',col,'LineWidth',1.5)

str1 = title1;

%str3 = 'Blue: 50-59% S+I';

t = {str1};

title(t, 'FontSize', 18, 'FontWeight', 'bold')

xlim([0 10]);

ylim([0 10]);

set(gca,'XTick',[0,1,2,3,4,5,6,7,8,9,10])

set(gca,'YTick',[0,1,2,3,4,5,6,7,8,9,10])

headings = {'XCoord','YCoord','DStart','DEnd','DLigand','VStart','VEnd',...

'VLigand','Sequence','%Total'};

xlswrite(title1,headings,1,'A1');

row=1;

xcoord={};

ycoord={};

DStart={};

DEnd={};

pepD={};

VStart={};

VEnd={};

pepV={};

seqnc={};

totalper={};

Dsort = MergeSortStructIdx(D);

D = Dsort;

for i=1:length(V)

k=1;

while k<=length(D)

j=1;

M=struct;

Max=struct;

permatch=[];

prcntl=[];

v1 = num2str(cell2mat(V(i).peptide));

v2 = num2str(cell2mat(D(k).peptide));

[~,~,persim,persame,seq] = Compare(v1,v2);

permatch(j) = persim + persame;

M(j).idxA = D(k).idxA;

M(j).idxB = D(k).idxB;

M(j).pertotal = persim + persame;

M(j).peptide = v2;

M(j).percentile = D(k).percentile;

M(j).seq = seq;

while j<3 && k<length(D) && D(k).idxA + 1 == D(k+1).idxA

j=j+1; k=k+1;

v2 = num2str(cell2mat(D(k).peptide));

[~,~,persim,persame,seq] = Compare(v1,v2);

permatch(j) = persim + persame;

M(j).idxA = D(k).idxA;

M(j).idxB = D(k).idxB;

M(j).pertotal = persim + persame;

M(j).peptide = v2;

M(j).percentile = D(k).percentile;

M(j).seq = seq;

end

permax = max(permatch);

q=1;

for m=1:length(M)

if M(m).pertotal == permax

Max(q).idxA = M(m).idxA;

Max(q).idxB = M(m).idxB;

Max(q).pertotal = M(m).pertotal;

Max(q).peptide = M(m).peptide;

Max(q).percentile = M(m).percentile;

Max(q).seq = M(m).seq;

prcntl(q) = M(m).percentile;

q=q+1;

end

end

smallest = min(prcntl);

n=1; a=0;

while n<=length(Max) && a==0

if Max(n).percentile == smallest

a=1; b=0; x=0;

if AA~='X'

for y=4:12

if ((Max(n).peptide(y)==AA) && (v1(y)~=AA)) || ...

((Max(n).peptide(y)~=AA) && (v1(y)==AA))

x=1;

end

end

end

if x==0

if permax >= 60

scatter(Max(n).percentile,V(i).percentile,'r', 'fill');

b=1;

elseif permax > 50

scatter(Max(n).percentile,V(i).percentile,'b');

b=1;

end

end

if b==1

xcoord{row,1} = Max(n).percentile;

ycoord{row,1} = V(i).percentile;

DStart{row,1} = Max(n).idxA;

DEnd{row,1} = Max(n).idxB;

pepD{row,1} = Max(n).peptide;

VStart{row,1} = V(i).idxA;

VEnd{row,1} = V(i).idxB;

pepV{row,1} = num2str(cell2mat(V(i).peptide));

seqnc{row,1} = Max(n).seq;

totalper{row,1} = permax;

row=row+1;

end

end

n=n+1;

end

k=k+1;

end

end

if ~isempty(xcoord)

xlswrite(title1,xcoord,1,'A2')

xlswrite(title1,ycoord,1,'B2')

xlswrite(title1,DStart,1,'C2')

xlswrite(title1,DEnd,1,'D2')

xlswrite(title1,pepD,1,'E2')

xlswrite(title1,VStart,1,'F2')

xlswrite(title1,VEnd,1,'G2')

xlswrite(title1,pepV,1,'H2')

xlswrite(title1,seqnc,1,'I2')

xlswrite(title1,totalper,1,'J2')

end

**Compare.m**

function [numsim,numsame,persim,persame,seq] = Compare(v1,v2)

seq = [];

v1 = upper(v1);

v2 = upper(v2);

numsim = 0;

numsame = 0;

n1 = length(v1);

n2 = length(v2);

n = min(n1,n2);

for p=1:n

if v1(p)==v2(p)

numsame = numsame + 1;

temp = upper(v1(p));

seq = [seq,temp];

elseif (((v1(p)=='D' && v2(p)=='E') || (v1(p)=='E' && v2(p)=='D')) || ...

((v1(p)=='R' && v2(p)=='K') || (v1(p)=='K' && v2(p)=='R')) || ...

((v1(p)=='A' && v2(p)=='V') || (v1(p)=='V' && v2(p)=='A')) || ...

((v1(p)=='A' && v2(p)=='L') || (v1(p)=='L' && v2(p)=='A')) || ...

((v1(p)=='A' && v2(p)=='I') || (v1(p)=='I' && v2(p)=='A')) || ...

((v1(p)=='A' && v2(p)=='M') || (v1(p)=='M' && v2(p)=='A')) || ...

((v1(p)=='V' && v2(p)=='L') || (v1(p)=='L' && v2(p)=='V')) || ...

((v1(p)=='V' && v2(p)=='I') || (v1(p)=='I' && v2(p)=='V')) || ...

((v1(p)=='V' && v2(p)=='M') || (v1(p)=='M' && v2(p)=='V')) || ...

((v1(p)=='L' && v2(p)=='I') || (v1(p)=='I' && v2(p)=='L')) || ...

((v1(p)=='L' && v2(p)=='M') || (v1(p)=='M' && v2(p)=='L')) || ...

((v1(p)=='I' && v2(p)=='M') || (v1(p)=='M' && v2(p)=='I')) || ...

((v1(p)=='Y' && v2(p)=='F') || (v1(p)=='F' && v2(p)=='Y')) || ...

((v1(p)=='S' && v2(p)=='T') || (v1(p)=='T' && v2(p)=='S')) || ...

((v1(p)=='N' && v2(p)=='Q') || (v1(p)=='Q' && v2(p)=='N')))

numsim = numsim + 1;

temp2 = lower(v1(p));

seq = [seq,temp2];

else

seq = [seq,'*'];

end

end

persim = numsim/n*100;

persame = numsame/n*100;

end

MergeStruct.m and MergeSortStructIdx.m adopted from Van Loan, Charles F. and K.-Y. Daisy Fan. Insight Through Computing: A MATLAB Introduction to Computational Science and Engineering (2010), Pages: 347 and 350
