# Supplementary material for: Predicting Hemagglutinin MHC-II Ligand Analogues in Anti-TNFα Biologics: Implications for Immunogenicity of Pharmaceutical Proteins
Source: PLoS One. 2015 Aug 13;10(8):e0135451. doi: 10.1371/journal.pone.0135451 (PMC4536234; doi:10.1371/journal.pone.0135451)
Supplement: S2 Fig — “*” indicates junctions between variable and constant regions in the polypeptide. Boxed regions indicate HA ligands that are mapped to biologic ligands (Table 3). (PDF) [file pone.0135451.s002.pdf]

```
Identities = 170/229 (74%), Positives = 203/229 (89%)
001  EVKLEESGGGLVQPGGSMKLSCVASGFIFSNHWMNWVRQSPEKGLEWVAEIRSKSINSATHYAE IFX HC
     ||:| |||||||||| |::|||:|||| |::: |:||||:| ||||||: | : : ::   ||:
001  EVQLVESGGGLVQPGRSLRLSCAASGFTFDDYAMHWVRQAPGKGLEWVSAI-TWN-SGHIDYAD ADA HC

                                                                *
065  SVKGRFTISRDDSKSAVYLQMTDLRTEDTGVYYCSR-NYY--GSTYDYWGQGTTLTVSSASTKG
     ||:||||||||::|:::||:|::||:|||:||||:: :|   :|: ||||||| :|||||||||
063  SVEGRFTISRDNAKNSLYLDMNSLRAEDTAVYYCAKVSYLSTASSLDYWGQGTLVTVSSASTKG
                                                                *

126  PSVFPLAPSSKSTSGGTAALGCLVKDYFPEPVTVSWNSGALTSGVHTFPAVLQSSGLYSLSSVV
     ||||||||||||||||||||||||||||||||||||||||||||||||||||||||||||||||
127  PSVFPLAPSSKSTSGGTAALGCLVKDYFPEPVTVSWNSGALTSGVHTFPAVLQSSGLYSLSSVV

190  TVPSSSLGTQTYICNVNHKPSNTKVDKKVEPKSCDKT
     ||||||||||||||||||||||||||||:
191  TVPSSSLGTQTYICNVNHKPSNTKVDKKI--------
```

```
Identities = 166/214 (78%), Positives = 195/214 (91%)
001  DILLTQSPAILSVSPGERVSFSCRASQFVGSSIHWYQQRTNGSPRLLIKYASESMSGIPSRFSG IFX LC
     || :||||: ||:| |:||:::||||| : : : ||||: : :|:|||  ||  :||:||||||
001  DIQMTQSPSSLSASVGDRVTITCRASQGIRNYLAWYQQKPGKAPKLLIYAASTLQSGVPSRFSG ADA LC

                                                   *
065  SGSGTDFTLSINTVESEDIADYYCQQSHSWPFTFGSGTNLEVKRTVAAPSVFIFPPSDEQLKSG
     |||||||||:|:::: ||:| ||||: :  |:|||:||::|:||||||||||||||||||||||
065  SGSGTDFTLTISSLQPEDVATYYCQRYNRAPYTFGQGTKVEIKRTVAAPSVFIFPPSDEQLKSG
                                                   *

129  TASVVCLLNNFYPREAKVQWKVDNALQSGNSQESVTEQDSKDSTYSLSSTLTLSKADYEKHKVY
     ||||||||||||||||||||||||||||||||||||||||||||||||||||||||||||||||
129  TASVVCLLNNFYPREAKVQWKVDNALQSGNSQESVTEQDSKDSTYSLSSTLTLSKADYEKHKVY

193  ACEVTHQGLSSPVTKSFNRGEC
     ||||||||||||||||||||
193  ACEVTHQGLSSPVTKSFNRGE-
```
