# Supplementary material for: Predicting Hemagglutinin MHC-II Ligand Analogues in Anti-TNFα Biologics: Implications for Immunogenicity of Pharmaceutical Proteins
Source: PLoS One. 2015 Aug 13;10(8):e0135451. doi: 10.1371/journal.pone.0135451 (PMC4536234; doi:10.1371/journal.pone.0135451)
Supplement: S3 Fig — The purple box in CA07-CA3726 enclosed the core sequence of B and T epitopes mapped to ADA HC CDR-3 (Fig 6). The core LSTASSWSY is broken in NY3095, NY1050, and PA10. Boxed regions in blue indicate common analogues, biologic sequences mapped to viral ligands (Table 3). Despite the >80% identities, cross-matching CA07 with ligands of the four strains (using MatchLig), S4 Fig (m-o) shows different patterns of ligands for all alleles except 0401 (with respect to their percentile rank in binding). (PDF) [file pone.0135451.s003.pdf]

```
Identities = 555/566 (98%), Positives = 565/566 (100%)
001  MKAILVVLLYTFATANADTLCIGYHANNSTDTVDTVLEKNVTVTHSVNLLEDKHNGKLCKLRGV CA07
     ||||||||||||||||||||||||||||||||||||||||||||||||||||||||||||||||
001  MKAILVVLLYTFATANADTLCIGYHANNSTDTVDTVLEKNVTVTHSVNLLEDKHNGKLCKLRGV CA3726

065  APLHLGKCNIAGWILGNPECESLSTASSWSYIVETPSSDNGTCYPGDFIDYEELREQLSSVSSF
     ||||||||||||||||||||||||||||||||||| |||||||||||||:||||||||||||||
065  APLHLGKCNIAGWILGNPECESLSTASSWSYIVETSSSDNGTCYPGDFINYEELREQLSSVSSF

129  ERFEIFPKTSSWPNHDSNKGVTAACPHAGAKSFYKNLIWLVKKGNSYPKLSKSYINDKGKEVLV
     |||||||||||||||||||||||||||||||||||||||||||||||||||:||||||||||||
129  ERFEIFPKTSSWPNHDSNKGVTAACPHAGAKSFYKNLIWLVKKGNSYPKLSQSYINDKGKEVLV

193  LWGIHHPSTSADQQSLYQNADAYVFVGSSRYSKKFKPEIAIRPKVRDQEGRMNYYWTLVEPGDK
     |||||||||:|||||||||||||||||:||||||||||||||||||||||||||||||||||||
193  LWGIHHPSTTADQQSLYQNADAYVFVGTSRYSKKFKPEIAIRPKVRDQEGRMNYYWTLVEPGDK

257  ITFEATGNLVVPRYAFAMERNAGSGIIISDTPVHDCNTTCQTPKGAINTSLPFQNIHPITIGKC
     ||||||||||||||||:||||||||||||||||||||||||||:||||||||||||||||||||
257  ITFEATGNLVVPRYAFTMERNAGSGIIISDTPVHDCNTTCQTPEGAINTSLPFQNIHPITIGKC

321  PKYVKSTKLRLATGLRNIPSIQSRGLFGAIAGFIEGGWTGMVDGWYGYHHQNEQGSGYAADLKS
     |||||||||||||||||:||||||||||||||||||||||||||||||||||||||||||||||
321  PKYVKSTKLRLATGLRNVPSIQSRGLFGAIAGFIEGGWTGMVDGWYGYHHQNEQGSGYAADLKS

385  TQNAIDEITNKVNSVIEKMNTQFTAVGKEFNHLEKRIENLNKKVDDGFLDIWTYNAELLVLLEN
     ||||||:|||||||||||||||||||||||||||||||||||||||||||||||||||||||||
385  TQNAIDKITNKVNSVIEKMNTQFTAVGKEFNHLEKRIENLNKKVDDGFLDIWTYNAELLVLLEN

449  ERTLDYHDSNVKNLYEKVRSQLKNNAKEIGNGCFEFYHKCDNTCMESVKNGTYDYPKYSEEAKL
     |||||||||||||||||||:||||||||||||||||||||||||||||||||||||||||||||
449  ERTLDYHDSNVKNLYEKVRNQLKNNAKEIGNGCFEFYHKCDNTCMESVKNGTYDYPKYSEEAKL

513  NREEIDGVKLESTRIYQILAIYSTVASSLVLVVSLGAISFWMCSNGSLQCRICI
     |||:||||||||||||||||||||||||||||||||||||||||||||||||||
513  NREKIDGVKLESTRIYQILAIYSTVASSLVLVVSLGAISFWMCSNGSLQCRICI
```

```
Identities = 451/566 (80%), Positives = 523/566 (92%)
001  MKAILVVLLYTFATANADTLCIGYHANNSTDTVDTVLEKNVTVTHSVNLLEDKHNGKLCKLRGV CA07
     ||: |:||| ||::: |||:||||||||||||||||||||||||||||||||::|||||| |:|:
001  MKVKLLVLLCTFTATYADTICIGYHANNSTDTVDTVLEKNVTVTHSVNLLENSHNGKLCLLKGI NY3095

065  APLHLGKCNIAGWILGNPECESLSTASSWSYIVETPSSDNGTCYPGDFIDYEELREQLSSVSSF
     |||:||:|::||||||||||| | :  ||||||| |: :||||||| | |||||||||||||||
065  APLQLGNCSVAGWILGNPECELLISKESWSYIVEKPNPENGTCYPGHFADYEELREQLSSVSSF

129  ERFEIFPKTSSWPNHDSNKGVTAACPHAGAKSFYKNLIWLVKKGNSYPKLSKSYINDKGKEVLV
     |||||||| |||||| :  ||:|:| | | :|||:||:||: |:: ||:||||| |:| |||||
129  ERFEIFPKESSWPNH-TVTGVSASCSHNGESSFYRNLLWLTGKNGLYPNLSKSYANNKEKEVLV

193  LWGIHHPSTSADQQSLYQNADAYVFVGSSRYSKKFKPEIAIRPKVRDQEGRMNYYWTLVEPGDK
     |||:||| : :||::||:: :||| | ||:||:|| |||| ||||||||||:||||||:|||| 
192  LWGVHHPPSISDQKTLYHTENAYVSVVSSHYSRKFTPEIAKRPKVRDQEGRINYYWTLLEPGDT

257  ITFEATGNLVVPRYAFAMERNAGSGIIISDTPVHDCNTTCQTPKGAINTSLPFQNIHPITIGKC
     | |||:|||::||||||: |: ||||| |::|:  |:: ||||:||||:||||||:||:|||:|
256  IIFEANGNLIAPRYAFALSRGFGSGIINSNAPMDKCDAKCQTPQGAINSSLPFQNVHPVTIGEC

321  PKYVKSTKLRLATGLRNIPSIQSRGLFGAIAGFIEGGWTGMVDGWYGYHHQNEQGSGYAADLKS
     ||||:|:|||::||||||||||||||||||||||||||||||||||||||||||||||||| ||
320  PKYVRSAKLRMVTGLRNIPSIQSRGLFGAIAGFIEGGWTGMVDGWYGYHHQNEQGSGYAADQKS

385  TQNAIDEITNKVNSVIEKMNTQFTAVGKEFNHLEKRIENLNKKVDDGFLDIWTYNAELLVLLEN
     |||||: ||||||||||||||||||||||||:||:|:|||||||||||:|||||||||||||||
384  TQNAINGITNKVNSVIEKMNTQFTAVGKEFNKLERRMENLNKKVDDGFIDIWTYNAELLVLLEN

449  ERTLDYHDSNVKNLYEKVRSQLKNNAKEIGNGCFEFYHKCDNTCMESVKNGTYDYPKYSEEAKL
     |||||:||||||||||||:|||||||||||||||||||||:: ||||||||||||||||||:||
448  ERTLDFHDSNVKNLYEKVKSQLKNNAKEIGNGCFEFYHKCNDECMESVKNGTYDYPKYSEESKL

513  NREEIDGVKLESTRIYQILAIYSTVASSLVLVVSLGAISFWMCSNGSLQCRICI
     |||:||||||||  :||||||||||||||||:||||||||||||||||||||||
512  NREKIDGVKLESMGVYQILAIYSTVASSLVLLVSLGAISFWMCSNGSLQCRICI
```

```
Identities = 453/566 (80%), Positives = 525/566 (93%)
001  MKAILVVLLYTFATANADTLCIGYHANNSTDTVDTVLEKNVTVTHSVNLLEDKHNGKLCKLRGV CA07
     ||: |:||| ||::: |||:||||||||||||||||||||||||||||||||:|||||| |:|:
001  MKVKLLVLLCTFTATYADTICIGYHANNSTDTVDTVLEKNVTVTHSVNLLEDSHNGKLCLLKGI NY1050

065  APLHLGKCNIAGWILGNPECESLSTASSWSYIVETPSSDNGTCYPGDFIDYEELREQLSSVSSF
     |||:||:|::||||||||||| | :  |||||||||: :||||||| | |||||||||||||||
065  APLQLGNCSVAGWILGNPECELLISKESWSYIVETPNPENGTCYPGYFADYEELREQLSSVSSF

129  ERFEIFPKTSSWPNHDSNKGVTAACPHAGAKSFYKNLIWLVKKGNSYPKLSKSYINDKGKEVLV
     |||||||| |||||| :  ||:|:| | :|||:||:||: |:: ||:||||| |:| |||||
129  ERFEIFPKESSWPNH-TVTGVSASCSHNGKSSFYRNLLWLTGKNGLYPNLSKSYANNKEKEVLV

193  LWGIHHPSTSADQQSLYQNADAYVFVGSSRYSKKFKPEIAIRPKVRDQEGRMNYYWTLVEPGDK
     |||:||| : :||::||:: :||| | ||:||::| |||| ||||||||||:||||||:||||
192  LWGVHHPPNIGDQRALYHTENAYVSVVSSHYSRRFTPEIAKRPKVRDQEGRINYYWTLLEPGDT

257  ITFEATGNLVVPRYAFAMERNAGSGIIISDTPVHDCNTTCQTPKGAINTSLPFQNIHPITIGKC
     | |||:|||::||:|||: |: ||||| |::|: :|:: ||||:||||:||||||:||:|||:|
256  IIFEANGNLIAPRFAFALSRGFGSGIITSNAPMDECDAKCQTPQGAINSSLPFQNVHPVTIGEC

321  PKYVKSTKLRLATGLRNIPSIQSRGLFGAIAGFIEGGWTGMVDGWYGYHHQNEQGSGYAADLKS
     ||||:|||||::||||||||||||||||||||||||||||||||||||||||||||||||| ||
320  PKYVRSTKLRMVTGLRNIPSIQSRGLFGAIAGFIEGGWTGMVDGWYGYHHQNEQGSGYAADQKS

385  TQNAIDEITNKVNSVIEKMNTQFTAVGKEFNHLEKRIENLNKKVDDGFLDIWTYNAELLVLLEN
     |||||: ||||||||||||||||||||||||:||:|:|||||||||||||||||||||||||||
384  TQNAINGITNKVNSVIEKMNTQFTAVGKEFNKLERRMENLNKKVDDGFLDIWTYNAELLVLLEN

449  ERTLDYHDSNVKNLYEKVRSQLKNNAKEIGNGCFEFYHKCDNTCMESVKNGTYDYPKYSEEAKL
     |||||:||||||||||||:|||||||||||||||||||||:: ||||||||||||||||||:||
448  ERTLDFHDSNVKNLYEKVKSQLKNNAKEIGNGCFEFYHKCNDECMESVKNGTYDYPKYSEESKL

513  NREEIDGVKLESTRIYQILAIYSTVASSLVLVVSLGAISFWMCSNGSLQCRICI
     |||:||||||||  :||||||||||||||||:||||||||||||||||||||||
512  NREKIDGVKLESMGVYQILAIYSTVASSLVLLVSLGAISFWMCSNGSLQCRICI
```

```
Identities = 451/566 (80%), Positives = 523/566 (92%)
001  MKAILVVLLYTFATANADTLCIGYHANNSTDTVDTVLEKNVTVTHSVNLLEDKHNGKLCKLRGV  CA07
     ||: |:||| ||::: |||:|||||||||||||||||||||||||||||||::|||||| |:|:
001  MKVKLLVLLCTFTATYADTICIGYHANNSTDTVDTVLEKNVTVTHSVNLLENSHNGKLCLLKGI  PA10

065  APLHLGKCNIAGWILGNPECESLSTASSWSYIVETPSSDNGTCYPGDFIDYEELREQLSSVSSF
     |||:||:|::||||||||||| | :  ||||||| |: :||||||| | |||||||||||||||
065  APLQLGNCSVAGWILGNPECELLISKESWSYIVEKPNPENGTCYPGHFADYEELREQLSSVSSF

129  ERFEIFPKTSSWPNHDSNKGVTAACPHAGAKSFYKNLIWLVKKGNSYPKLSKSYINDKGKEVLV
     |||||||| |||||| :  ||:|:| | | :|||:||:||: |:: ||:||||| |:| |||||
129  ERFEIFPKESSWPNH-TVTGVSASCSHNGESSFYRNLLWLTGKNGLYPNLSKSYANNKEKEVLV

193  LWGIHHPSTSADQQSLYQNADAYVFVGSSRYSKKFKPEIAIRPKVRDQEGRMNYYWTLVEPGDK
     |||:||| : :||::||:: :||| | ||:||:|| |||| ||||||||||:||||||:||||
192  LWGVHHPPNIGDQKALYHTENAYVSVVSSHYSRKFTPEIAKRPKVRDQEGRINYYWTLLEPGDT

257  ITFEATGNLVVPRYAFAMERNAGSGIIISDTPVHDCNTTCQTPKGAINTSLPFQNIHPITIGKC
     | |||:|||::||||||: |: ||||| |::|:  |:: ||||:||||:||||||:||:|||:|
256  IIFEANGNLIAPRYAFALSRGFGSGIINSNAPMDKCDAKCQTPQGAINSSLPFQNVHPVTIGEC

321  PKYVKSTKLRLATGLRNIPSIQSRGLFGAIAGFIEGGWTGMVDGWYGYHHQNEQGSGYAADLKS
     ||||:|:|||::||||||||||||||||||||||||||||||||||||||||||||||||| ||
320  PKYVRSAKLRMVTGLRNIPSIQSRGLFGAIAGFIEGGWTGMVDGWYGYHHQNEQGSGYAADQKS

385  TQNAIDEITNKVNSVIEKMNTQFTAVGKEFNHLEKRIENLNKKVDDGFLDIWTYNAELLVLLEN
     |||||: ||||||||||||||||||||||||:||:|:|||||||||||:|||||||||||||||
384  TQNAINGITNKVNSVIEKMNTQFTAVGKEFNKLERRMENLNKKVDDGFIDIWTYNAELLVLLEN

449  ERTLDYHDSNVKNLYEKVRSQLKNNAKEIGNGCFEFYHKCDNTCMESVKNGTYDYPKYSEEAKL
     |||||:||||||||||||:|||||||||||||||||||||:: ||||||||||||||||||:||
448  ERTLDFHDSNVKNLYEKVKSQLKNNAKEIGNGCFEFYHKCNDECMESVKNGTYDYPKYSEESKL

513  NREEIDGVKLESTRIYQILAIYSTVASSLVLVVSLGAISFWMCSNGSLQCRICI
     |||:||||||||  :||||||||||||||||:||||||||||||||||||||||
512  NREKIDGVKLESMGVYQILAIYSTVASSLVLLVSLGAISFWMCSNGSLQCRICI
```
