# Supplementary material for: Predicting Hemagglutinin MHC-II Ligand Analogues in Anti-TNFα Biologics: Implications for Immunogenicity of Pharmaceutical Proteins
Source: PLoS One. 2015 Aug 13;10(8):e0135451. doi: 10.1371/journal.pone.0135451 (PMC4536234; doi:10.1371/journal.pone.0135451)
Supplement: S4 Fig — Predicted ligands placed along the polypeptides of (a) IFX, (b) ADA, and (c) ETN. Analogues in pre-2009 H1N1 influenza HA for five HLA-DR1 alleles: (d) IFX heavy chain presented by alleles associated with RA, (e) and not associated with RA; (f) IFX light chain presented by alleles associated with RA, (g) and not associated with RA; (h) ADA heavy chain presented by alleles associated with RA, (i) and not associated with RA; (j) ADA light chain presented by alleles associated with RA, (k) and not associated with RA; (l) ETN presented by alleles associated with RA, (m) and not associated with RA (j). (n), (o), and (p) show cross-matching of ligands between four HA sequences against CA07. P d denotes percentile ranking of a given ligand in a biologic, and P v denotes percentile ranking of a given ligand in a viral HA sequence. (PPTX) [file pone.0135451.s004.pptx]

## Slide 1
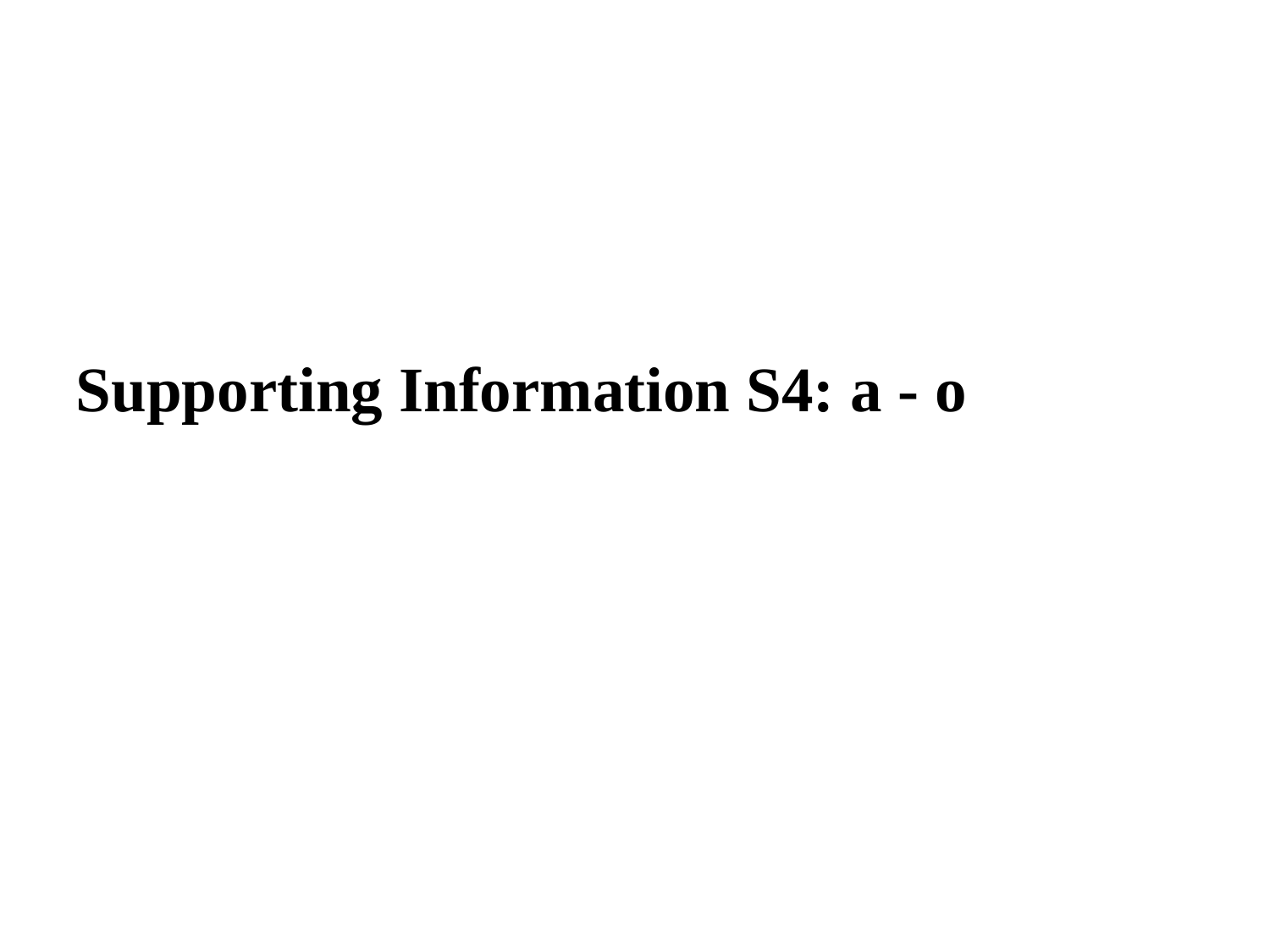

# Supporting Information S4: a - o

## Slide 2
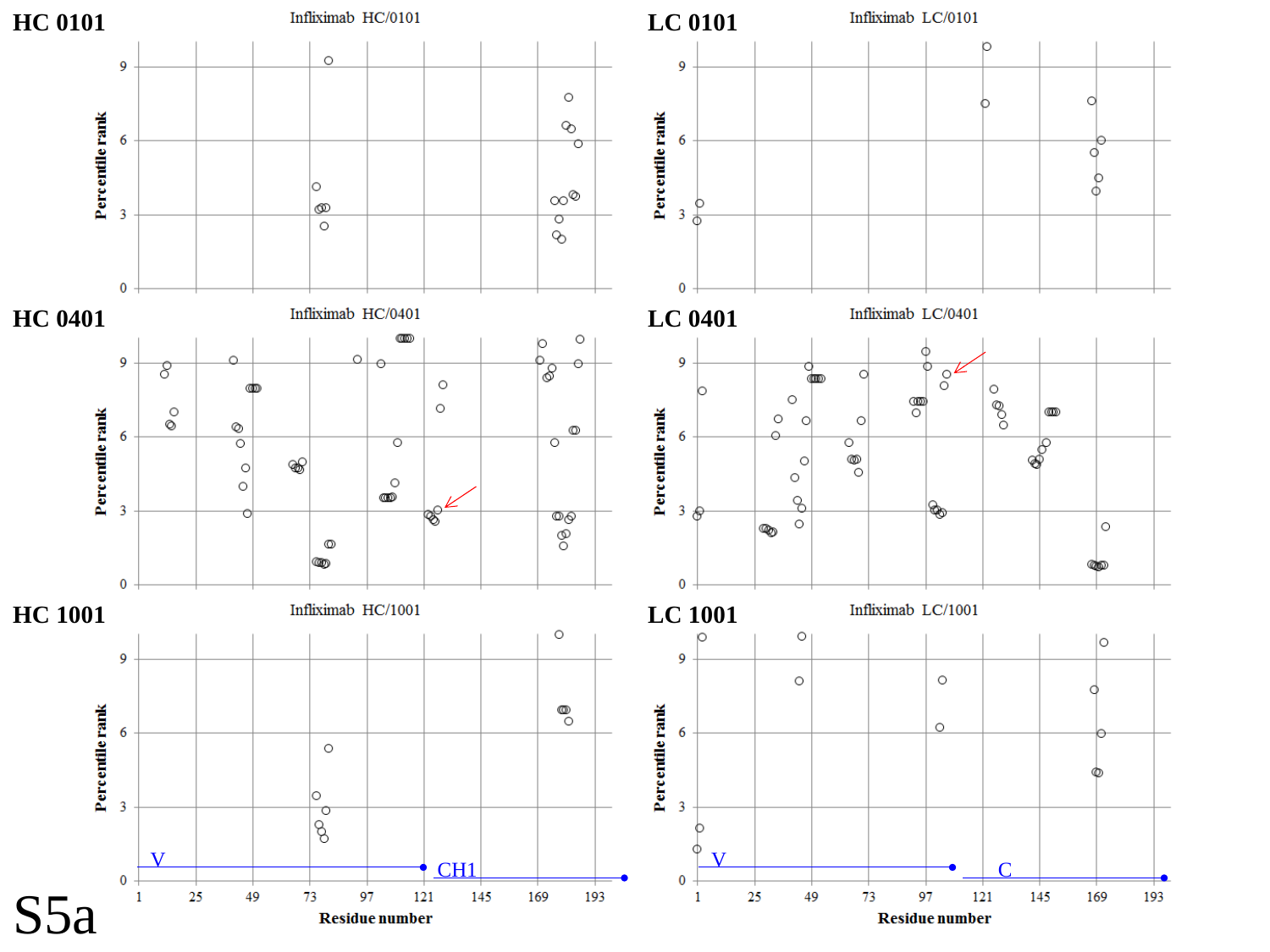

HC 0101
LC 0101
HC 0401
LC 0401
HC 1001
LC 1001
V
V
CH1
C
S5a

## Slide 3
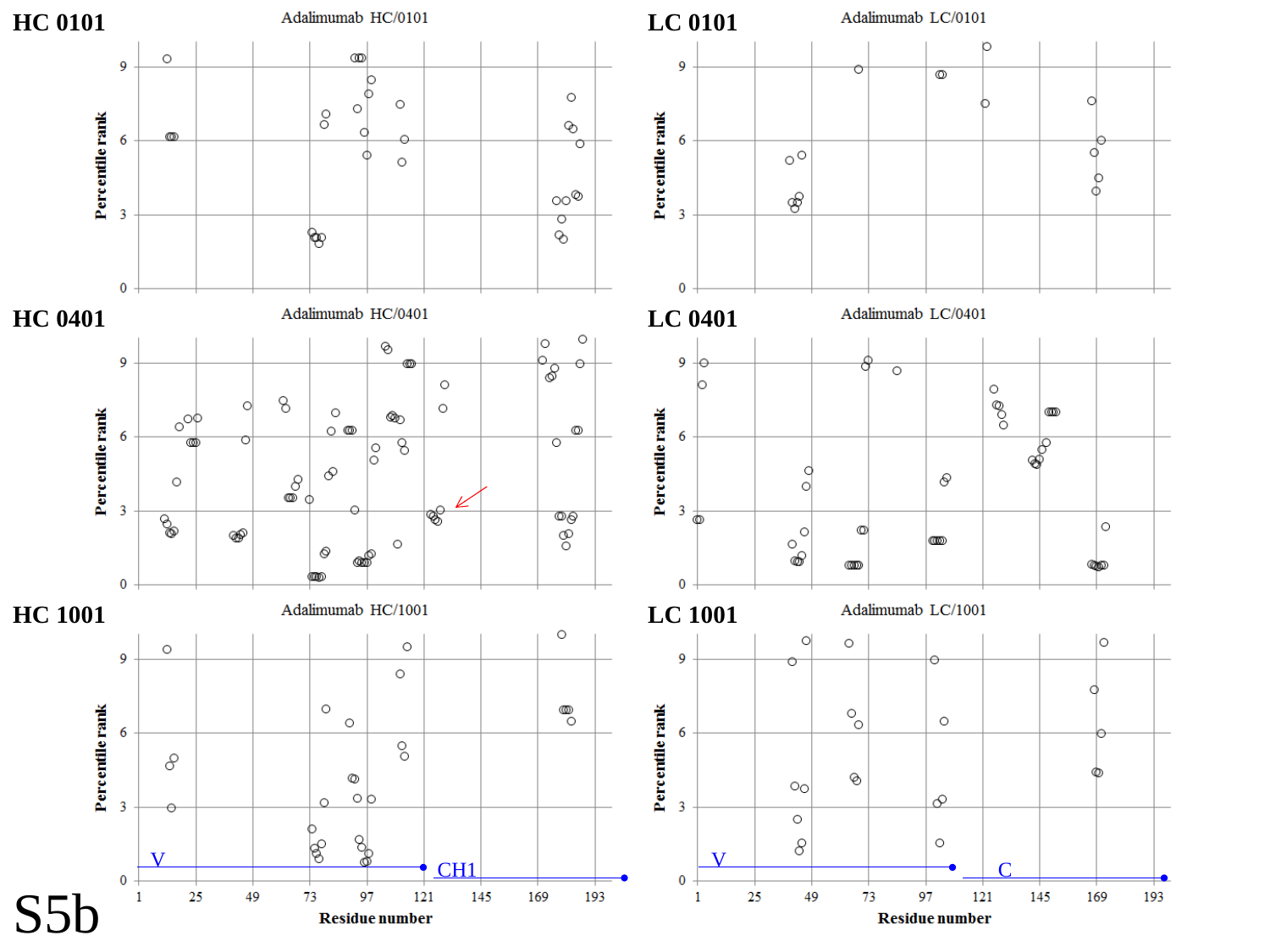

HC 0101
LC 0101
HC 0401
LC 0401
HC 1001
LC 1001
V
V
CH1
C
S5b

## Slide 4
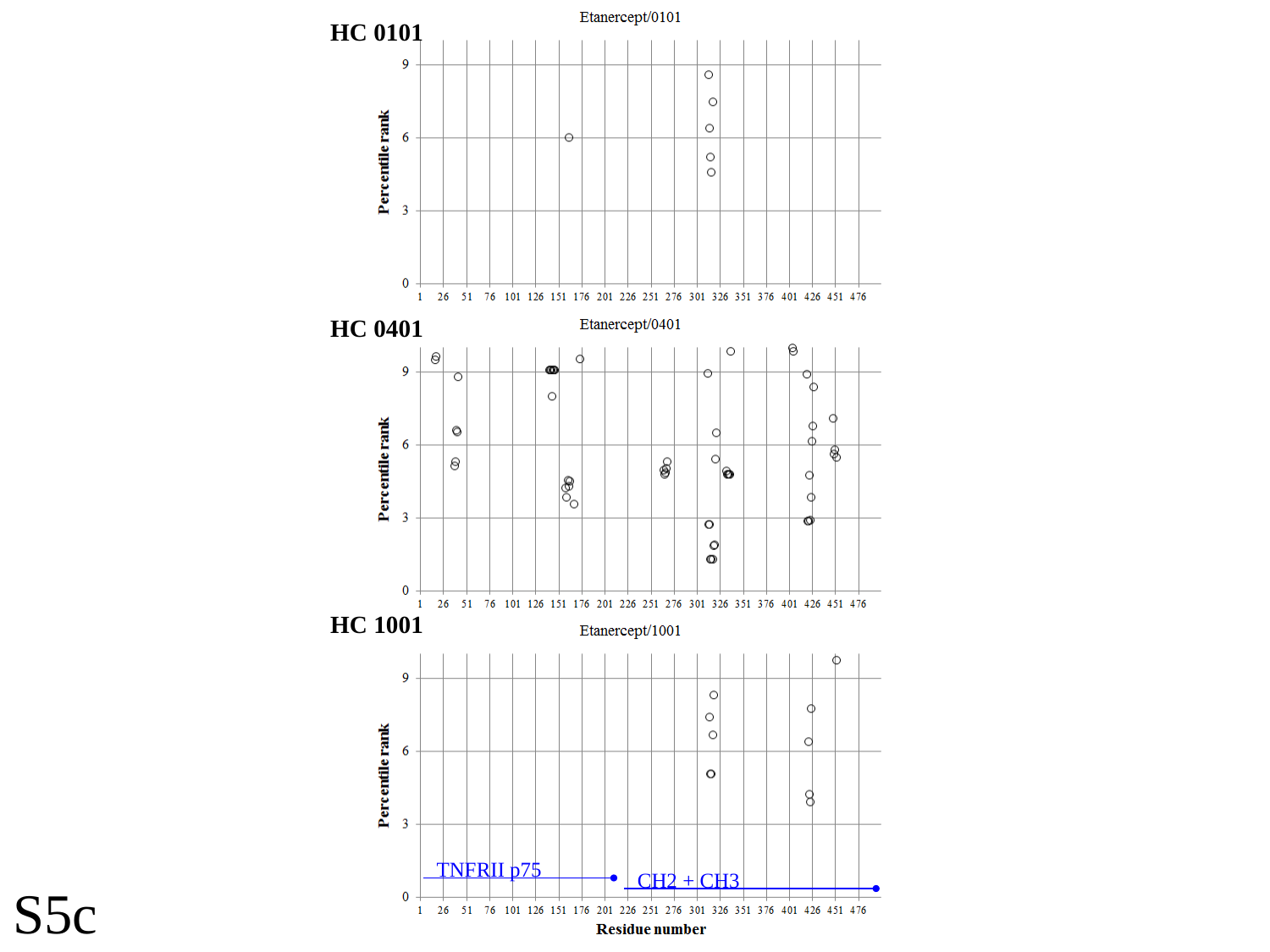

HC 0101
HC 0401
HC 1001
TNFRII p75
CH2 + CH3
S5c

## Slide 5
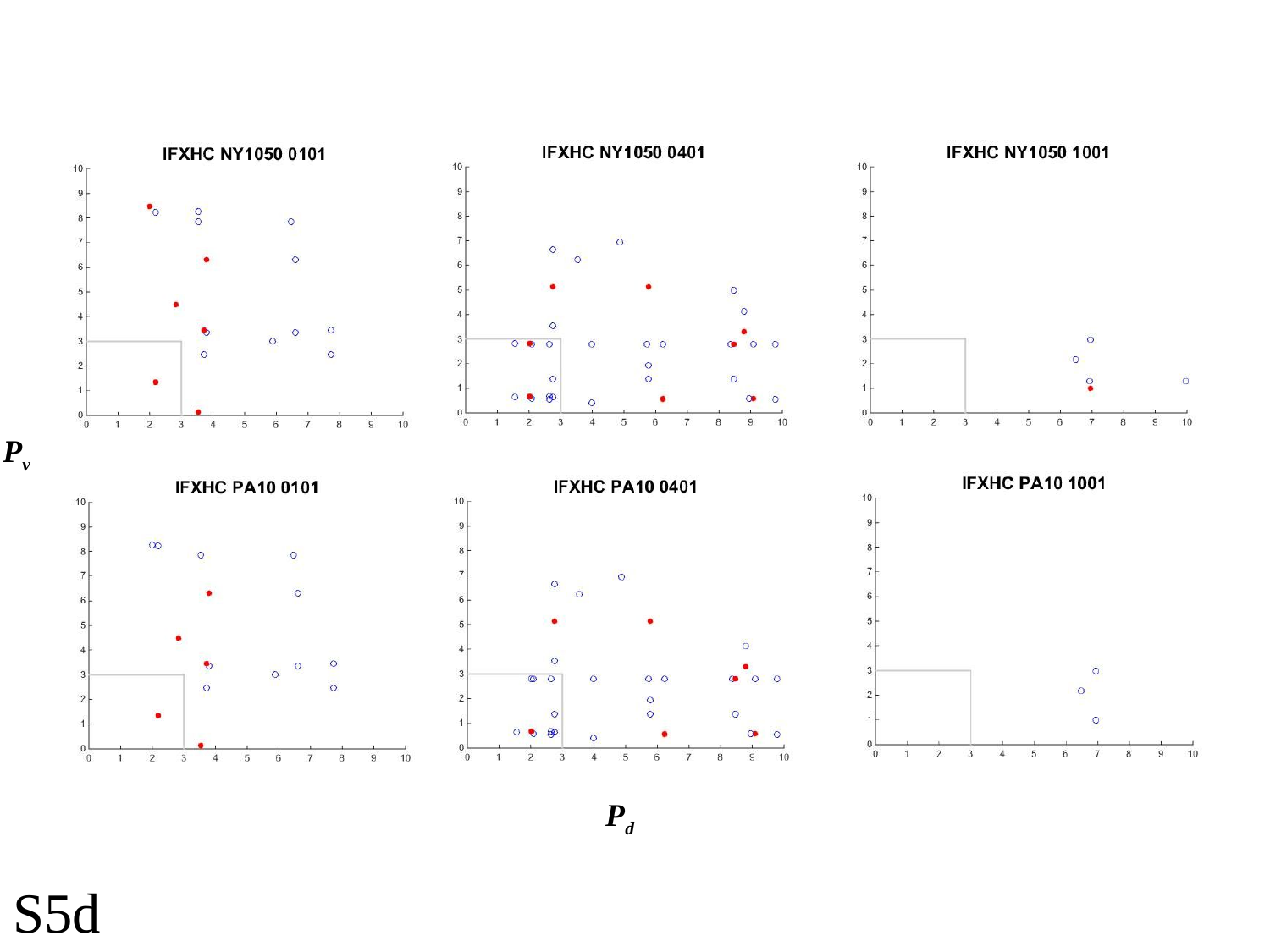

Pv
Pd
S5d

## Slide 6
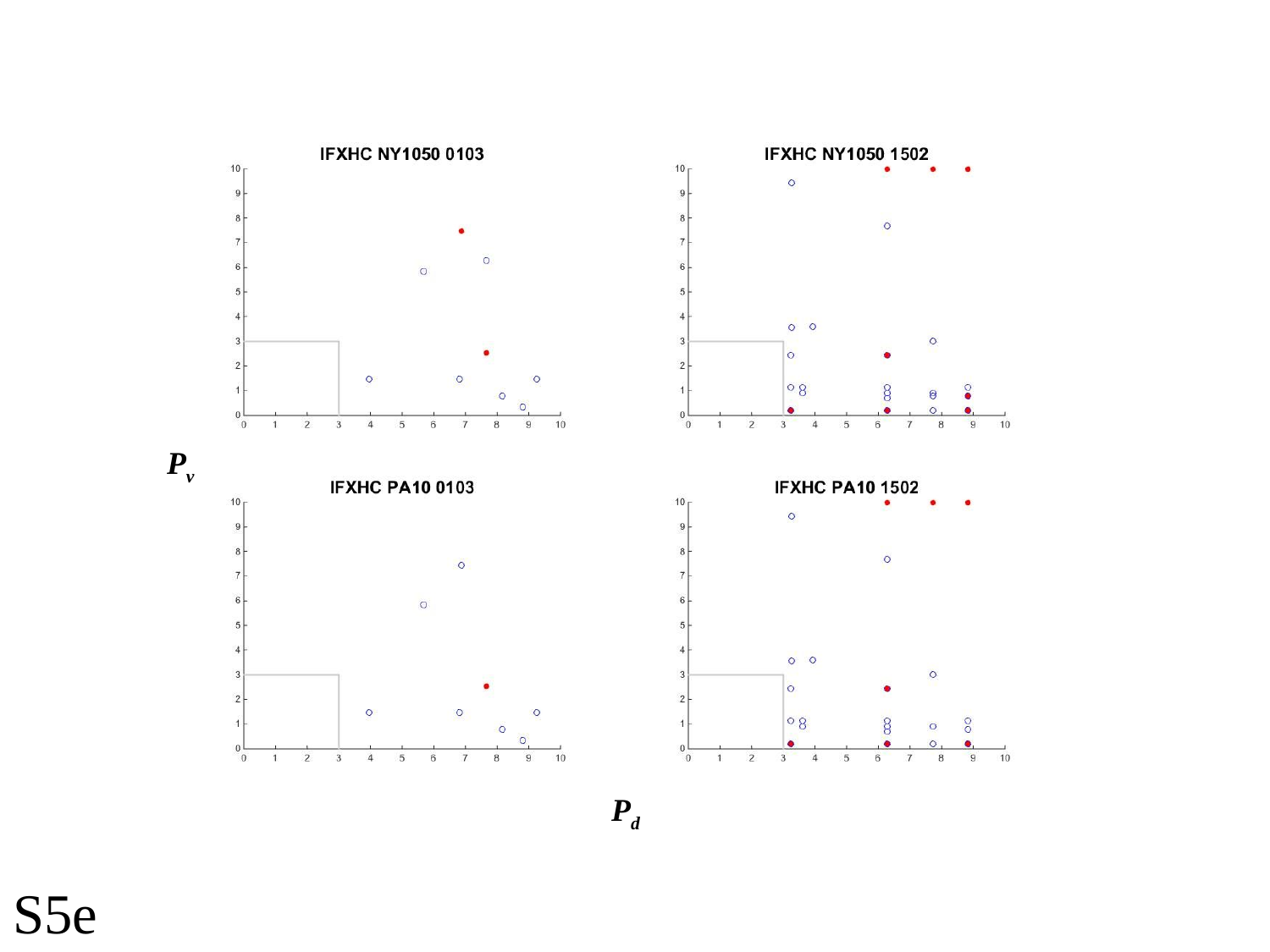

Pv
Pd
S5e

## Slide 7
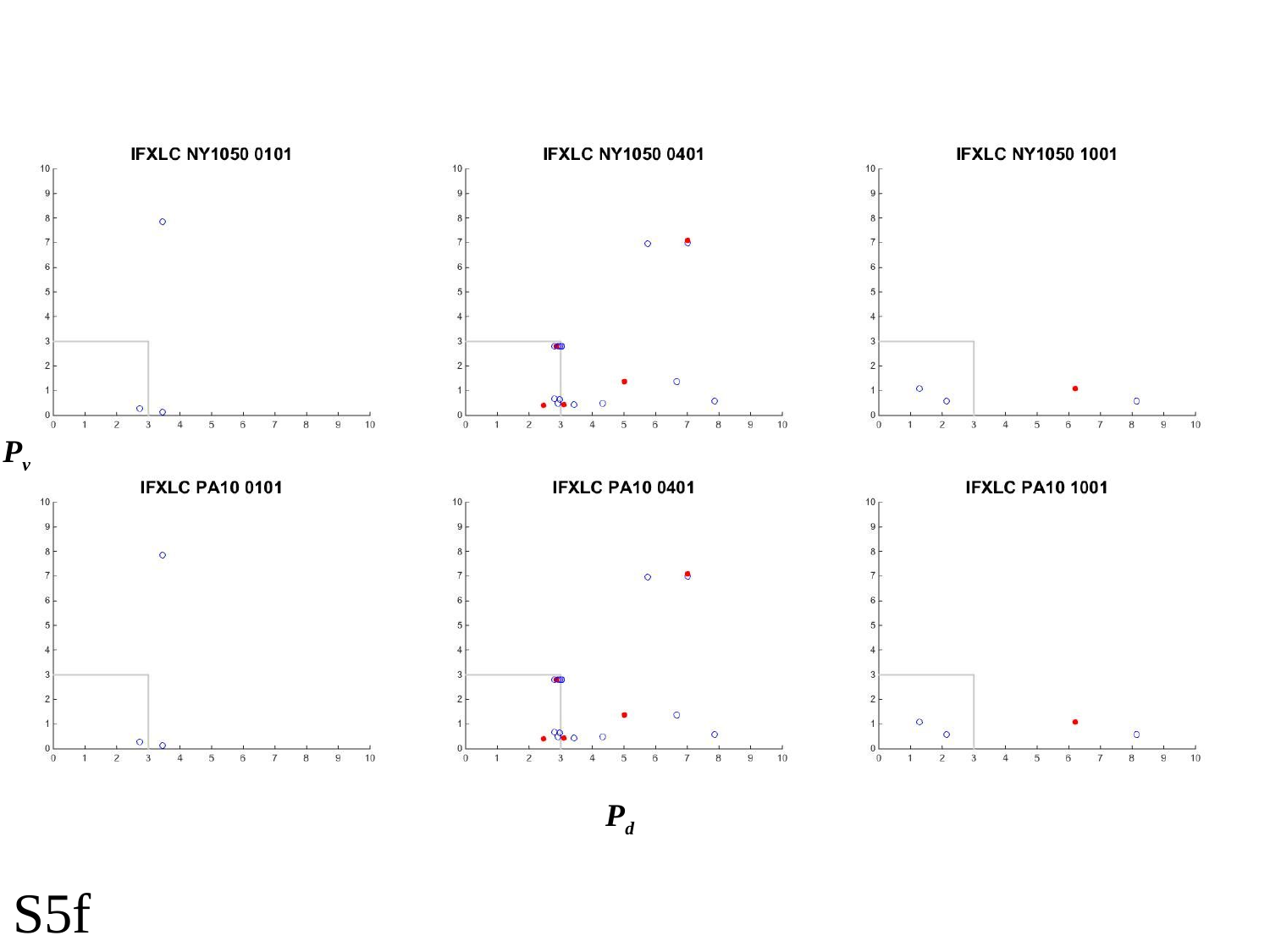

Pv
Pd
S5f

## Slide 8
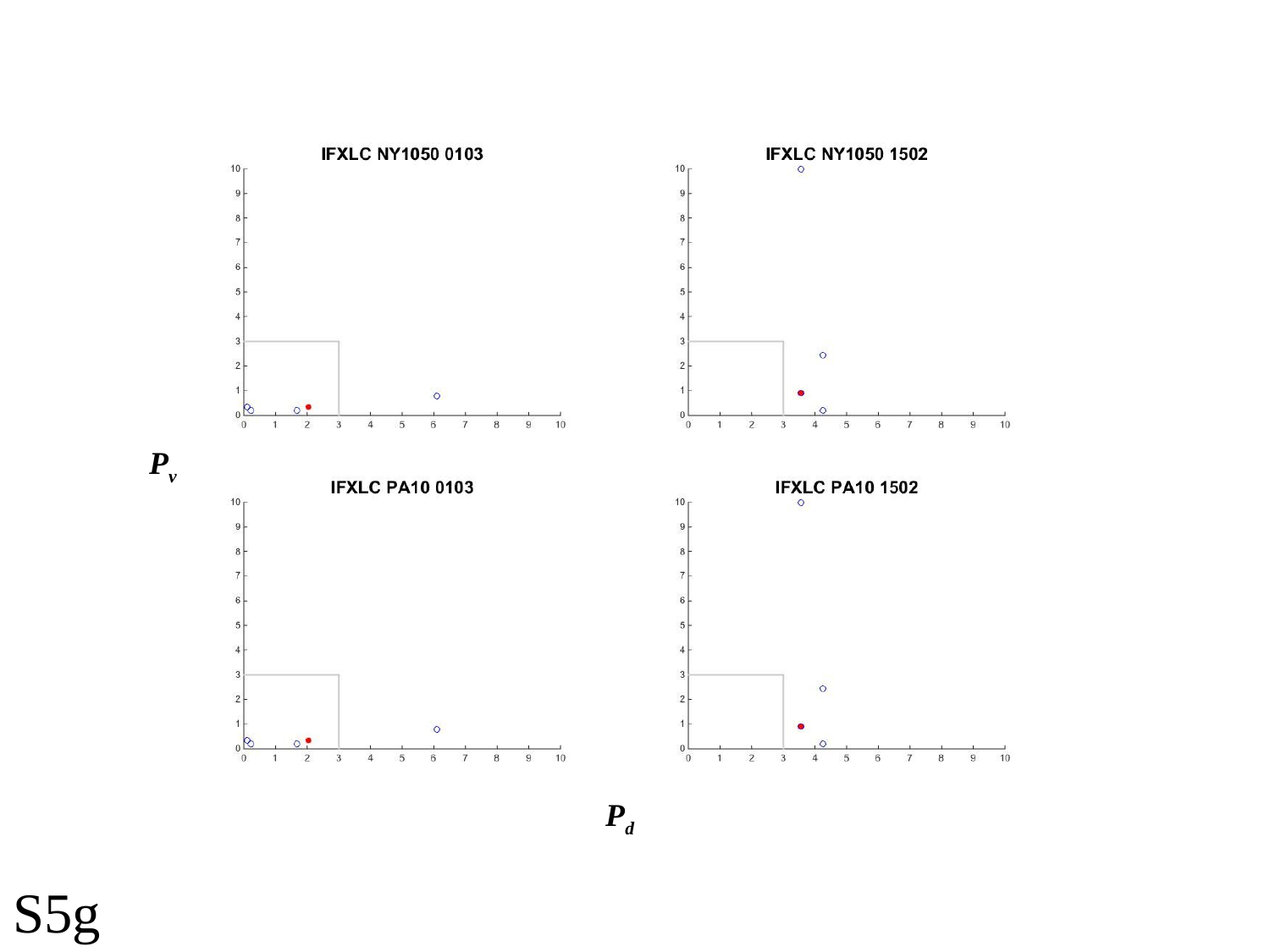

Pv
Pd
S5g

## Slide 9
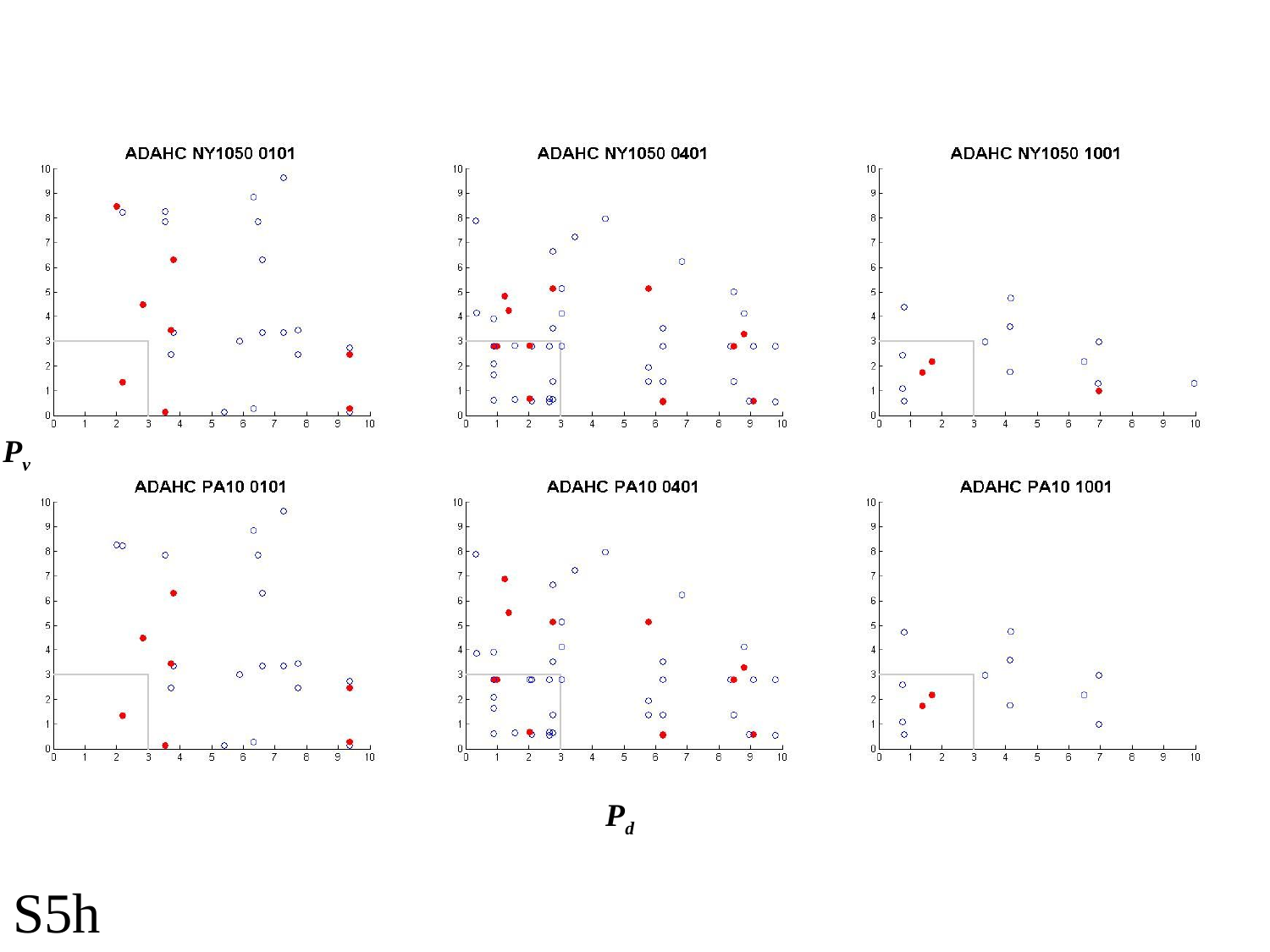

Pv
Pd
S5h

## Slide 10
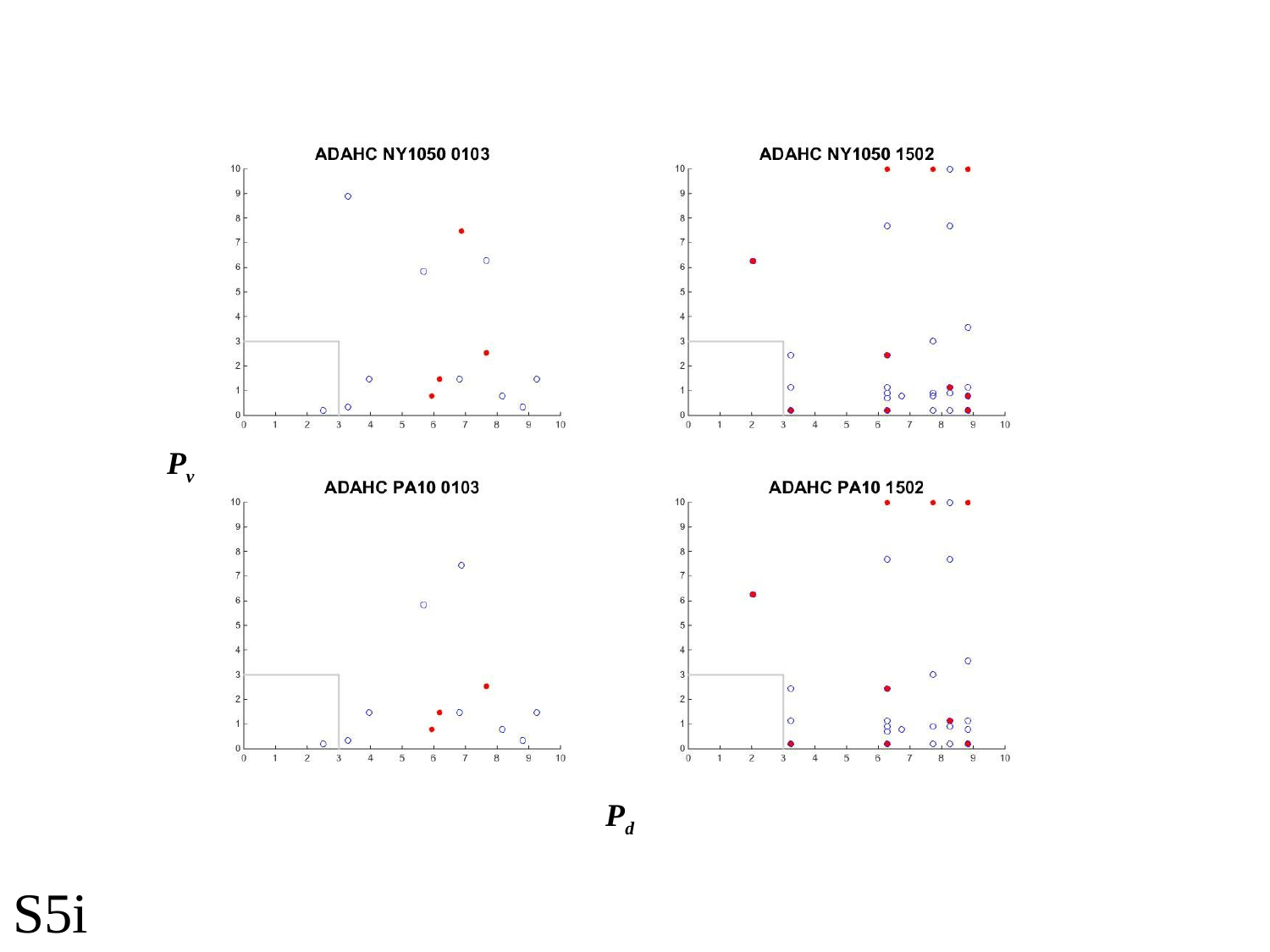

Pv
Pd
S5i

## Slide 11
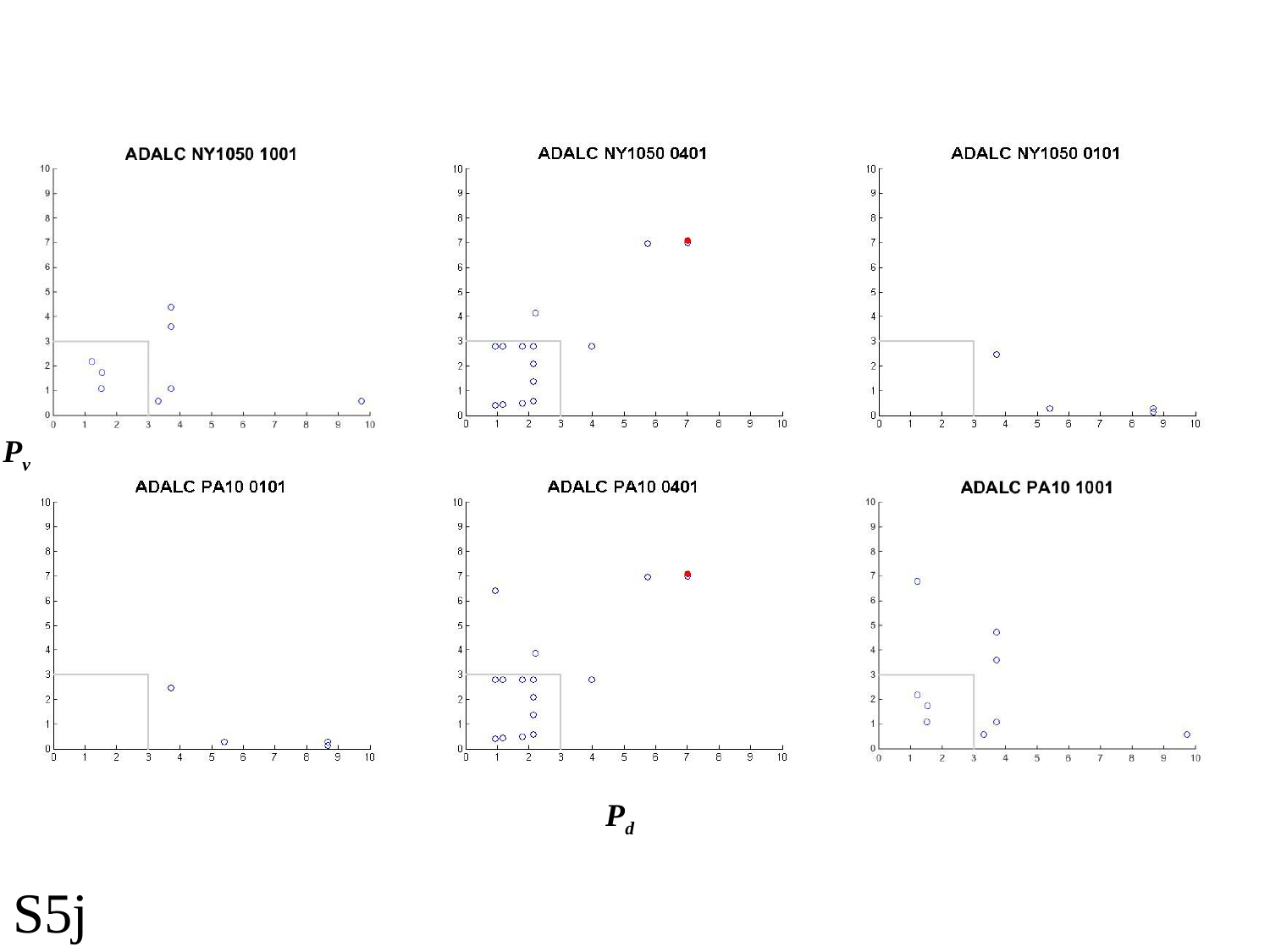

Pv
Pd
S5j

## Slide 12
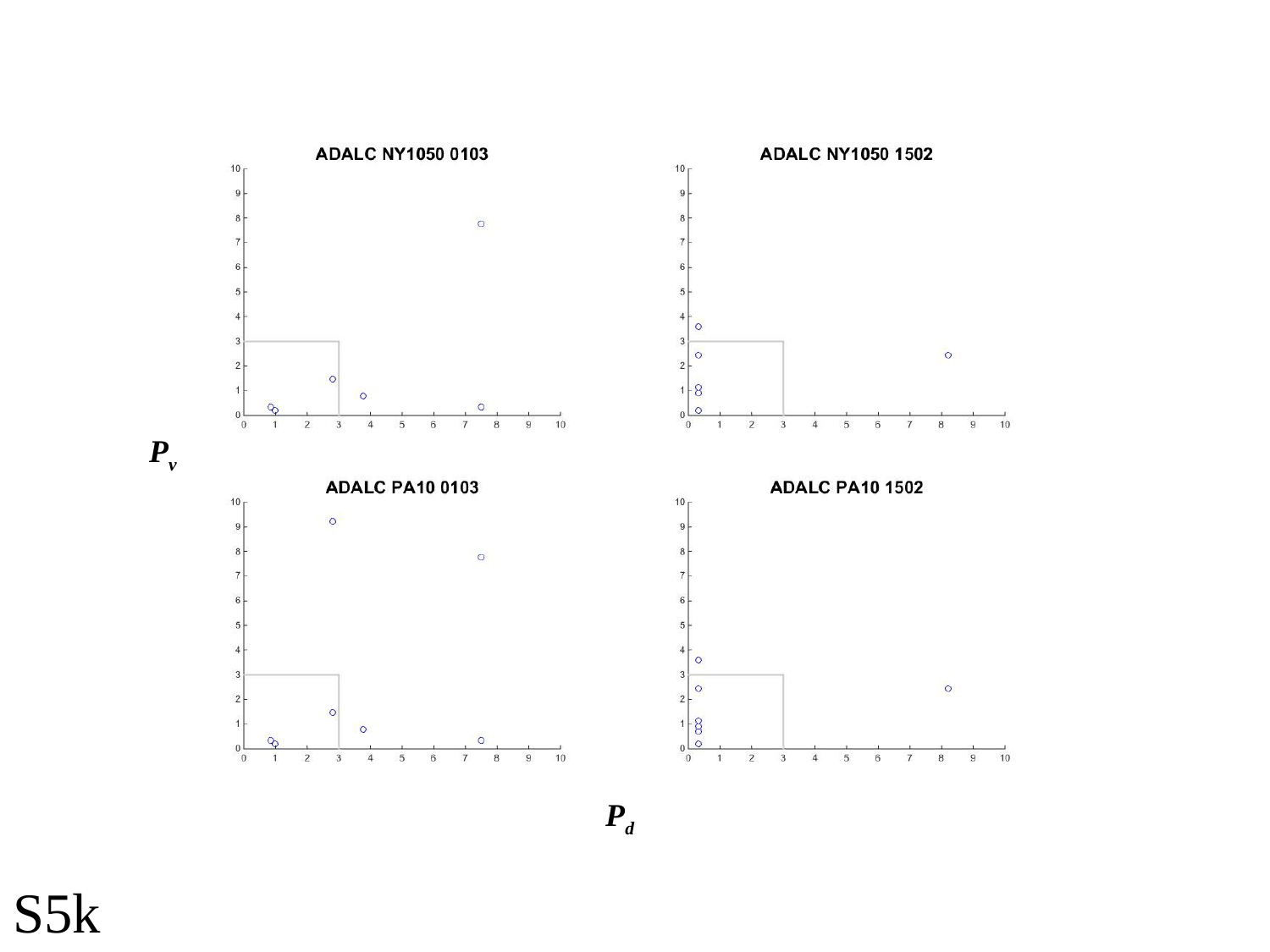

Pv
Pd
S5k

## Slide 13
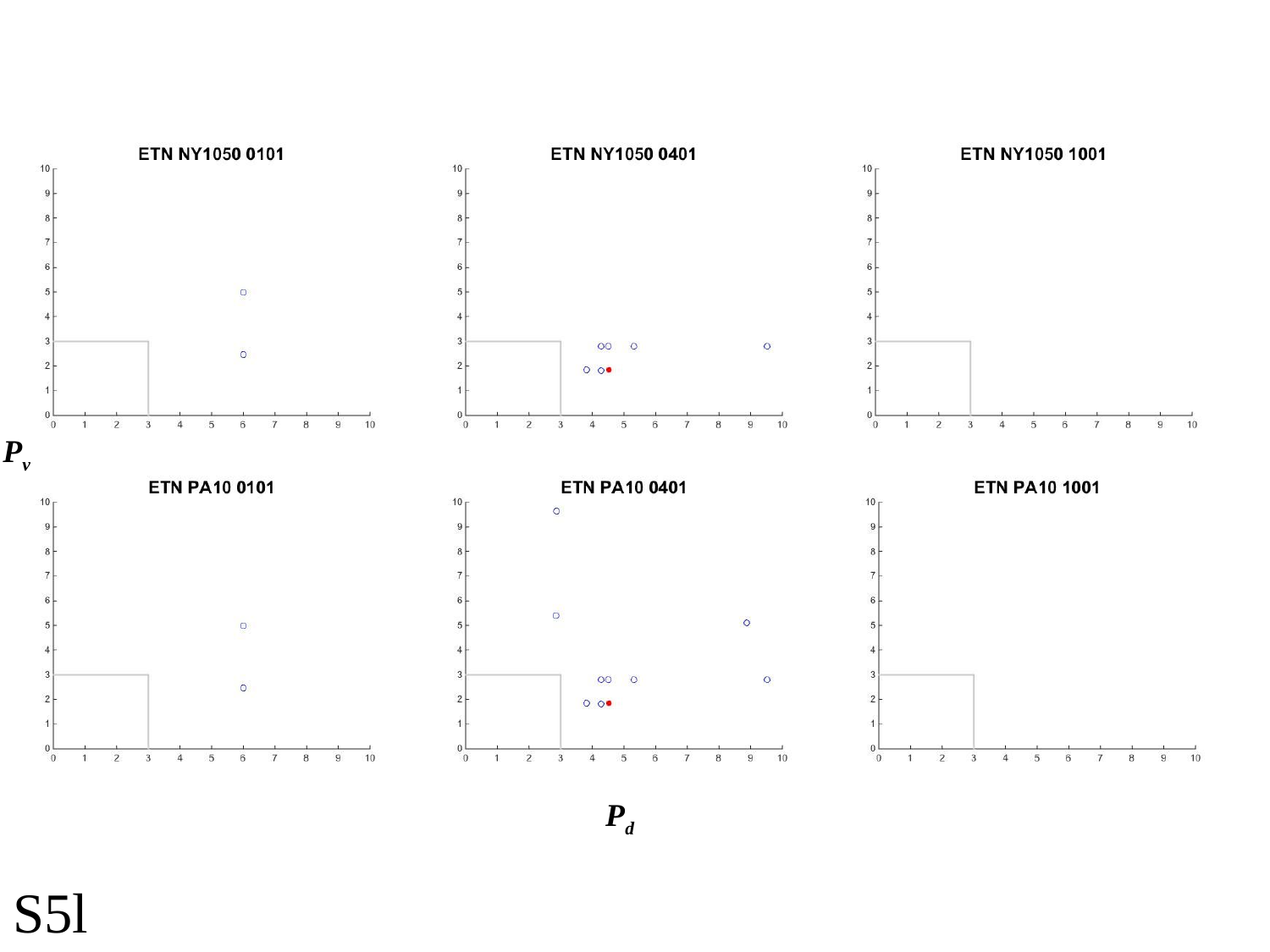

Pv
Pd
S5l

## Slide 14
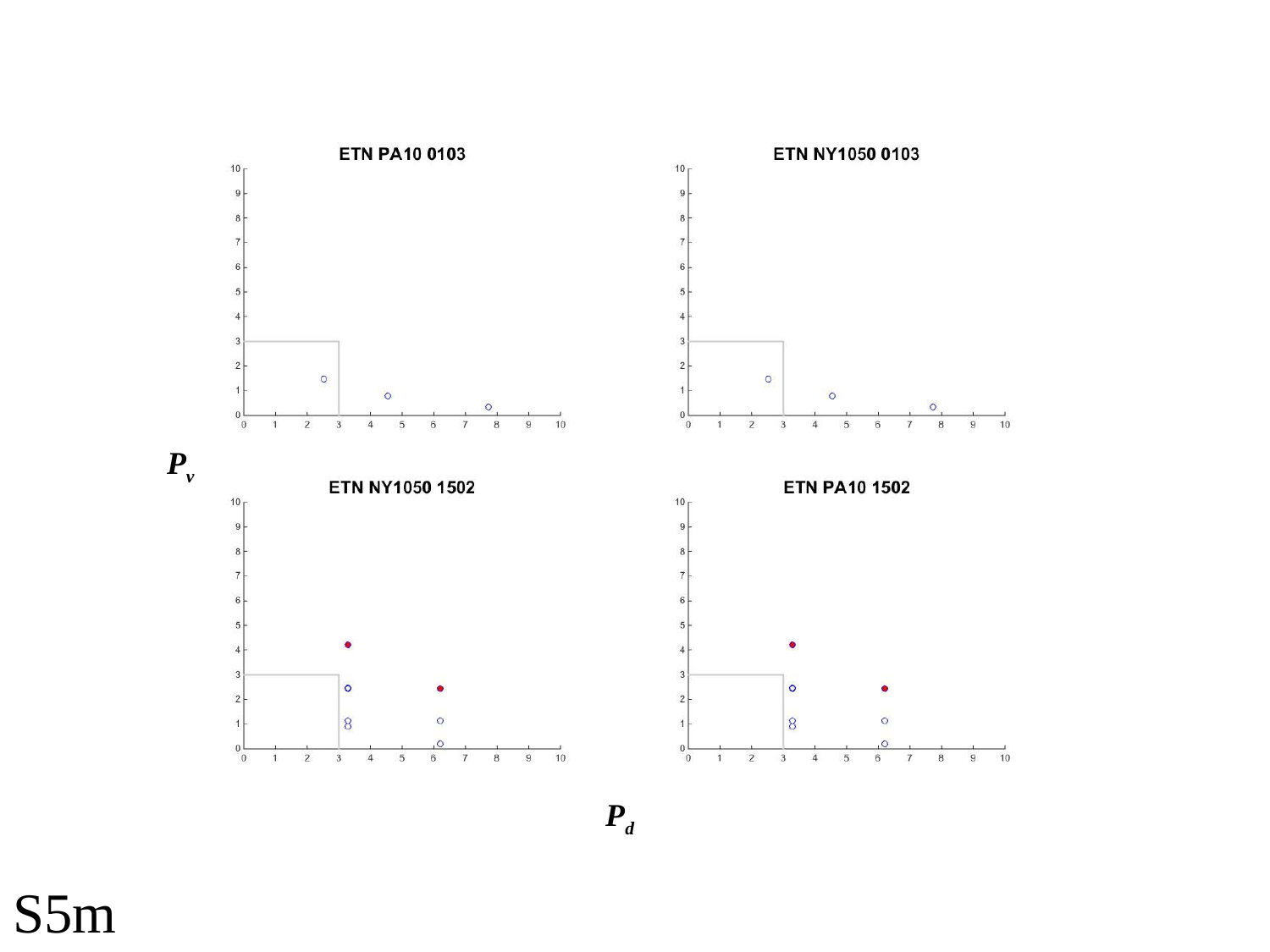

Pv
Pd
S5m

## Slide 15
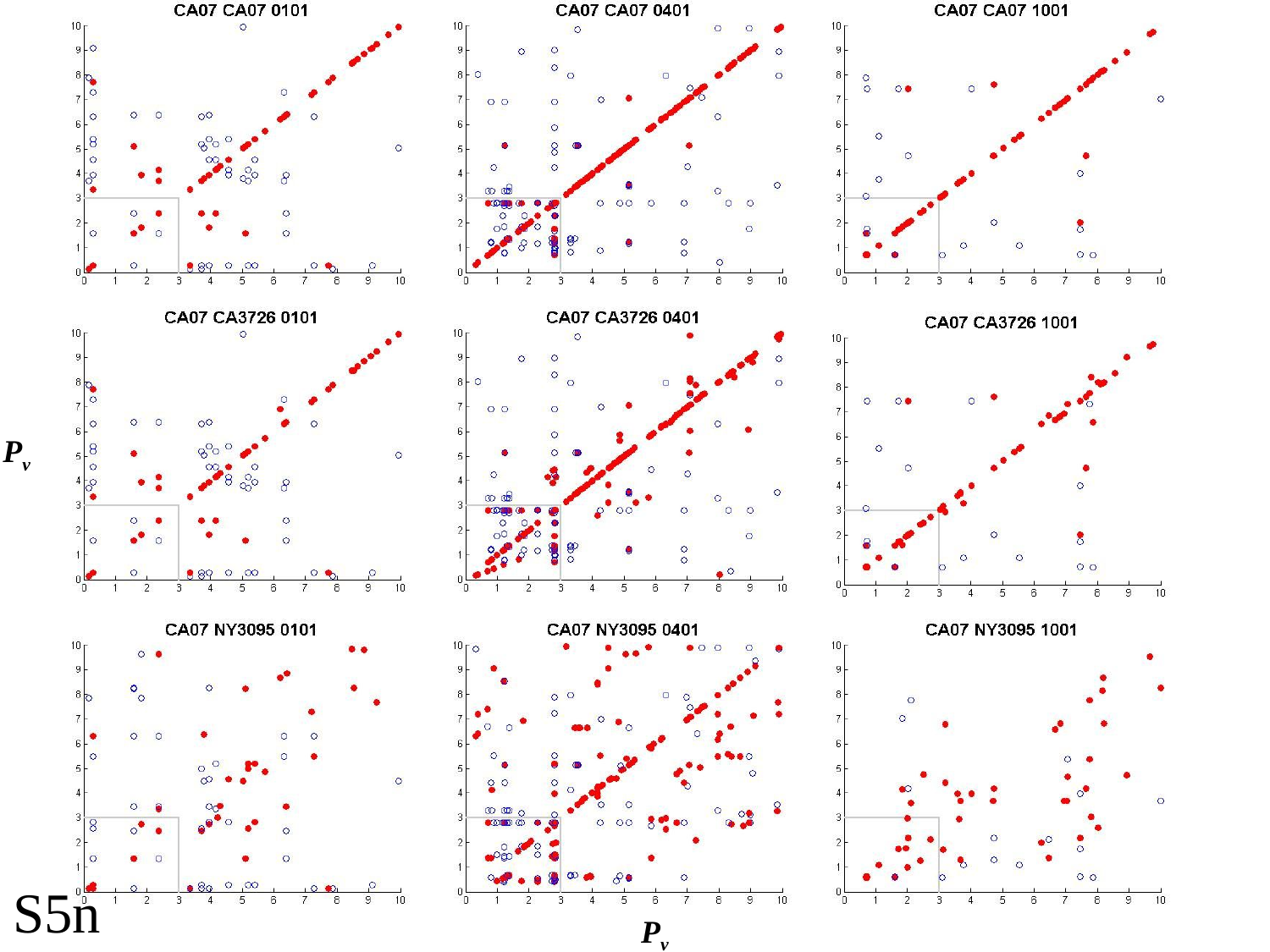

Pv
S5n
Pv

## Slide 16
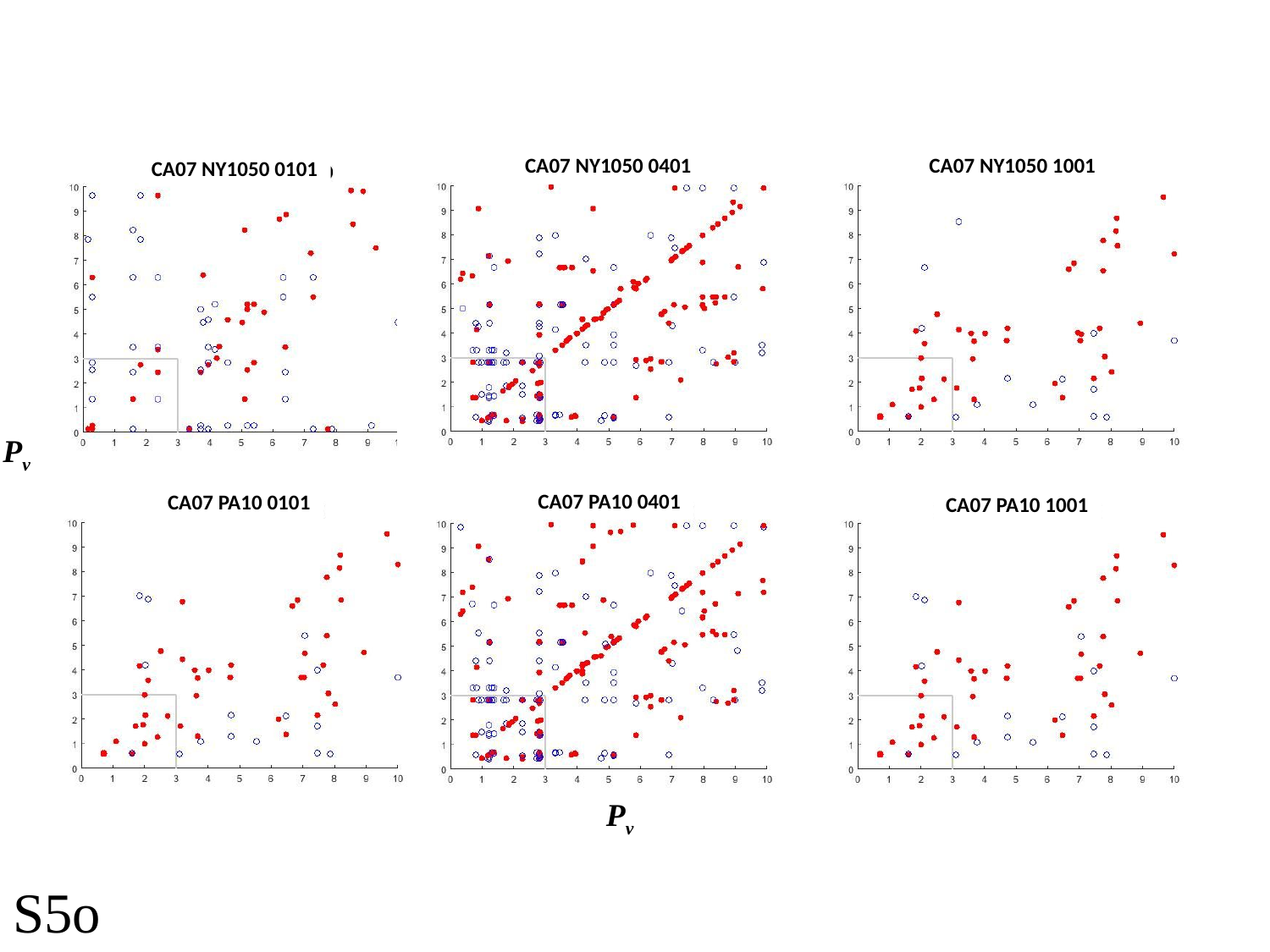

CA07 NY1050 1001
CA07 NY1050 0401
CA07 NY1050 0101
Pv
CA07 PA10 0401
CA07 PA10 0101
CA07 PA10 1001
Pv
S5o

## Slide 17
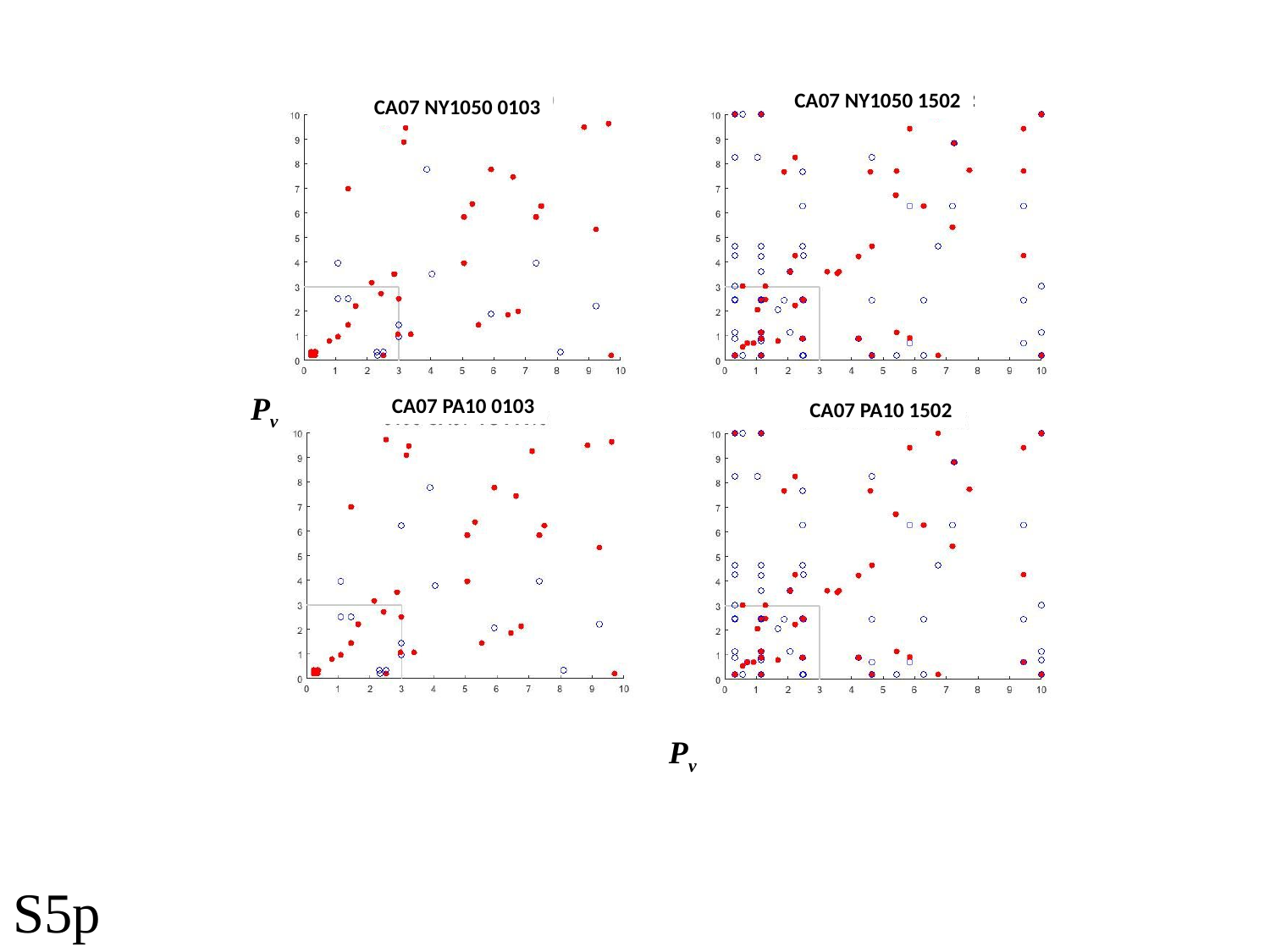

CA07 NY1050 1502
CA07 NY1050 0103
Pv
CA07 PA10 0103
CA07 PA10 1502
Pv
S5p
